# Quantum machine learning for predicting anastomotic leak: a clinical study

**DOI:** 10.1038/s41598-026-44402-x

**Published:** 2026-05-19

**Authors:** Vojtěch Novák, Ivan Zelinka, Lenka Přibylová, Lubomír Martínek, Vladimír Benčurik

**Affiliations:** 1 Department of Computer Science, VSB-Technical University of Ostrava, Ostrava, Czech Republic; 2 IT4Innovations National Supercomputing Center, Ostrava, Czech Republic; 3 Department of Applied Mathematics, Technical University of Ostrava, Ostrava, Czech Republic; 4 Department of Surgical Studies, Faculty of Medicine, University of Ostrava, Ostrava, Czech Republic; 5 Department of Surgery, University Hospital Ostrava, Ostrava, Czech Republic; 6 Surgical Department, Hospital AGEL Novy Jicin, Novy Jicin, Czech Republic

**Keywords:** Statistics, Computer science

## Abstract

Anastomotic leak is a life-threatening complication following colorectal surgery. This study benchmarks Quantum Neural Networks (QNNs) against hyperparameter-tuned classical models (logistic regression, multi-layer perceptrons, boosting algorithms) for anastomotic leak prediction. Using a 200-patient clinical dataset strictly bounded by a priori medical constraints, we simulated QNNs with ZZFeatureMap encoding and EfficientSU2/RealAmplitudes ansatze under realistic hardware noise. To ensure statistical reliability, performance metrics were averaged across 10 independent optimization runs. The EfficientSU2-BFGS configuration achieved the highest mean AUC of $0.797 \pm 0.024$, while RealAmplitudes with CMA-ES maximized Average Precision ($0.504 \pm 0.121$). Crucially, at a fixed, clinically necessary sensitivity of $83\%$, specific QNN configurations achieved significantly higher specificity (up to $66\%$) and Negative Predictive Value (up to $96\%$) compared to classical models (maximum $44\%$ and $94\%$, respectively), effectively minimizing false positives. However, classical models maintained superior probability calibration for continuous risk stratification. We conclude that QNNs offer robust discriminative performance for clinical screening, warranting further validation on larger, independent cohorts.

## Introduction

Anastomotic leak is a serious and potentially life-threatening complication arising from surgical procedures involving anastomosis, such as bowel resection, where two ends of a bowel are surgically connected. Anastomotic leak occurs when the connection fails to heal, resulting in leakage of contents into the abdominal cavity. In the case of bowel surgery, this can lead to peritonitis, sepsis, and other severe outcomes. Numerous risk factors, including smoking, malnutrition, immunosuppression, and prolonged operation times, have been associated with an increased likelihood of anastomotic leak. Despite advancements in surgical techniques and perioperative care, accurately predicting and managing the risk of anastomotic leak remains a critical challenge.

In this context, leveraging statistical and machine learning methodologies to identify key risk factors and develop predictive models is crucial for improving patient outcomes. ML offers the potential to process large datasets and uncover complex patterns that traditional statistical approaches might miss. By integrating these techniques, clinicians can better stratify patient risk, tailor surgical and postoperative care, and ultimately reduce the incidence and severity of anastomotic leak. The incorporation of ML and, potentially, QML for such predictive modeling represents an innovative step toward addressing this pressing medical issue.

A recent study by Benčurík et al.^1^ showed how intraoperative tools, like fluorescence angiography with indocyanine green (ICG), can significantly reduce anastomotic leak rates in rectal cancer surgery. Their data identified key risk factors such as diabetes and the use of transanal drains. Building on these findings, our work goes beyond standard data analysis by working directly with surgical experts to ensure the variables we use are clinically meaningful. By combining this medical insight with advanced predictive modeling, we aim to build a tool that actually fits the needs of a clinical environment and helps doctors make better decisions for their patients.

To achieve this level of robust, clinically actionable predictive modeling, advanced computational paradigms must be explored. While classical Machine Learning (ML) has firmly established itself as a cornerstone of artificial intelligence across diverse healthcare domains^2,3^, the field of Quantum Computing (QC) has concurrently witnessed rapid expansion. Despite current limitations that arise from Noisy Intermediate-Scale Quantum (NISQ) devices^4^, there is a potential for QC to outperform classical computers in select and highly complex ML applications^5^.

Quantum Machine Learning (QML)^6^ represents an intersection of quantum physics and ML, facilitating new interdisciplinary approaches^7^. Harnessing QML methodologies not only enhances performance but also accelerates data processing on QC platforms^8,9^. However, the efficacy of modern machine learning is fundamentally bounded by the polynomial computing time^10^, necessitating the simplification of quantum algorithms to yield reliable results. Within the domain of QML, four distinct approaches emerge based on the nature of data and the processing device, be it classical or quantum^11^.

Supervised learning tasks, such as those undertaken by Support Vector Machines (SVM)^12^, have undergone extensive exploration across various datasets, showcasing superior performance particularly with kernel-based methodologies^13^. Among quantum paradigms, the Variational Quantum Classifier (VQC) has garnered significant attention, particularly for classification tasks on NISQ devices^14^. Notably, a plenty of approaches exists for categorizing well-established supervised QML algorithms, including QSVM and VQC. Further strides have been made in this arena with the advent of quantum-inspired neural networks^15^, coupled with innovative applications such as hybridized low-depth VQC classification methods integrated with simple error-mitigation strategies^16^. Additionally, pre-processing techniques like Principle Component Analysis (PCA)^17^ have been leveraged, culminating in marked enhancements in categorization performance.

In the NISQ era, QML offers new computational possibilities within the constraints of contemporary hardware that typically provides 50 to 1000 noisy qubits, where deep quantum circuits remain challenging to execute due to noise and decoherence^18^. The introduction of parameterized quantum circuits enabled significant advancements, leading to influential algorithms like the Quantum Approximate Optimization Algorithm^19^ and the Variational Quantum Eigensolver^20^. These variational quantum algorithms use parameterized quantum circuits optimized through classical techniques, providing a robust framework for diverse problems through hybrid quantum-classical optimization loops^21^. In QML contexts, these circuits are termed quantum neural networks due to their machine learning applications^22,23^, leveraging quantum systems’ representational capabilities for supervised learning^24^.

Current research focuses on improving the performance and scalability of these hybrid models^25–27^. Whether QML algorithms can consistently outperform classical counterparts remains an open question^28–30^. Nevertheless, quantum circuit design has progressed considerably with architectures including hardware-efficient circuits^31^, the quantum alternating operator ansatz^32^, and dissipative quantum neural networks^33^. Data re-uploading techniques have also shown promise for encoding classical data into quantum models^34^. While these algorithms face challenges from stochastic quantum measurements and noise, advancing quantum hardware increasingly demonstrates their practical potential for real-world applications^24^.

In healthcare, biomedical engineering plays a crucial role in disease prediction and classification^35,36^. QML is increasingly integrated into domains such as medical healthcare records, which combine clinical test results, imaging data, and treatment histories^37,38^. Models including quantum support vector machines and variational quantum classifiers have demonstrated effectiveness in classifying complex conditions like diabetes and ischemic heart disease^39,40^. Studies show that these optimized hybrid quantum neural networks achieve high classification accuracies, highlighting the potential of QML in real-time clinical applications.

Furthermore, QML is rapidly transforming other biomedical domains like omics and biomedical imaging. Approaches using quantum optimization algorithms have been utilized for classifying gene subsets, which is particularly relevant in cancer detection^41–43^. In imaging, hybrid quantum-classical convolutional neural networks have been applied to COVID-19 diagnosis using X-ray images^44–47^ and show promising results in classifying malignant lesions in CT scans^48^. As quantum hardware advances, these techniques are expected to further enhance disease diagnosis, treatment optimization, and clinical decision-making.

While prior QML studies have explored classification on benchmark datasets^49–53^, its application to complex, noisy, and highly imbalanced real-world clinical problems like anastomotic leak prediction remains nascent. This study establishes its novelty in three key areas: It presents one of the first applications of QNNs to anastomotic leak prediction, a problem space dominated by classical statistical methods. It introduces a uniquely robust comparative framework, where QNNs simulated under realistic noise are benchmarked against hyperparameter-optimized classical models, with QNN performance validated across 10 independent runs to ensure statistical reliability. It moves beyond performance metrics to investigate QNN interpretability through experimental perturbation-based methods, offering a direct comparison to the feature importance derived from traditional clinical models like logistic regression.

## Materials and methods

**Fig. 1:**
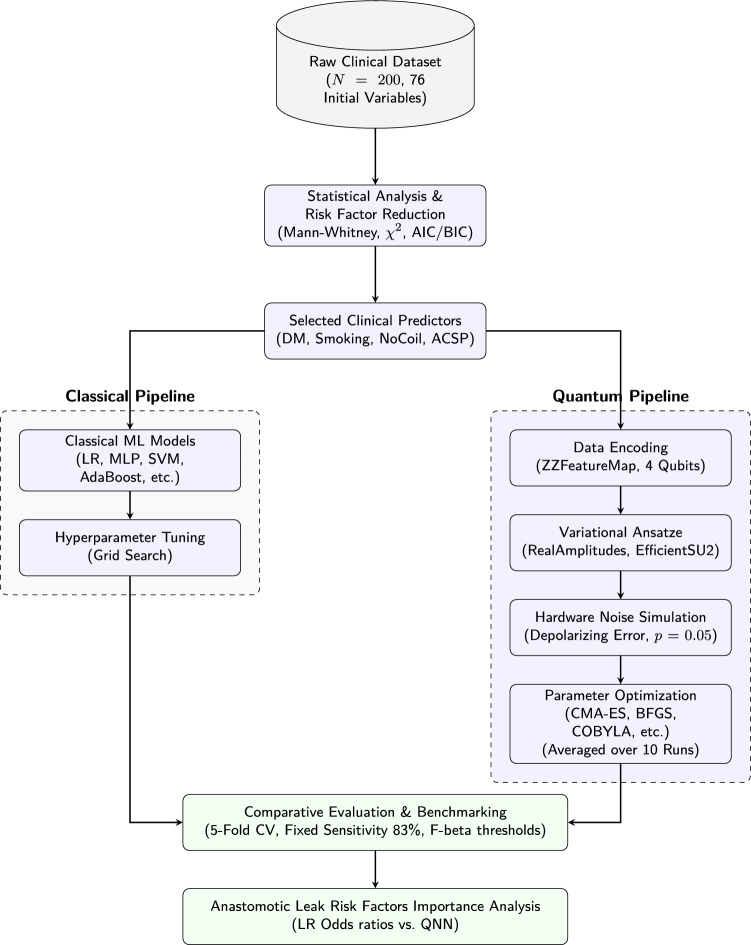
Workflow of the proposed methodology, detailing the end-to-end pipeline from clinical data preprocessing and risk factor selection to the parallel evaluation of classical models and noisy quantum neural networks, concluding with performance benchmarking and risk factor importance analysis.

This section details the experimental pipeline utilized to predict anastomotic leak outcomes and identify risk factors. We first introduce the medical problem in detail, define the clinical cohort, and outline all patient variables, including surgical techniques, patient history, and physiological markers with their respective units. Prior to any machine learning tasks, we perform rigorous statistical analyses and feature reduction to isolate the most significant clinical predictors. Building on this clinical foundation, we introduce the quantum machine learning methodology, detailing the design of our parameterized quantum circuits using specific data encoding maps and variational ansatze that we compare with classical machine learning models (Fig. 1).

### Problem description

Anastomotic leak is a severe complication following colorectal surgeries, particularly low rectal resections with total mesorectal excision for cancer. When treating malignant or large benign tumors in the colorectal area, the affected section of the intestine must be removed, and the two remaining ends are surgically reconnected. If the anastomotic site does not heal properly, it can rupture, leading to anastomotic leak. This complication is associated with significant morbidity and mortality, occurring in approximately 14% of cases in our dataset and contributing to up to 40% of surgery-related deaths. Identifying risk factors for anastomotic leak and developing predictive models are essential for improving patient outcomes.

Our study utilizes data collected from the Surgical Department of Hospital Nový Jičín a.s. between 2015 and 2016, comprising 200 patients (28 with an anastomotic leak, 172 without). The dataset includes 76 explanatory variables, categorized into two main groups: intraoperative techniques and patient history variables. The primary goal of this study was to identify the statistical significance of intraoperative techniques such as NoCoil, ACSP, PERFB, and ICG, and to identify potential risk factors for anastomotic leak occurrence. Furthermore, we aimed to establish if we could predict anastomotic leak from the significant factors associated with its occurrence. We will particularly focus on smoking status (Smoking), Diabetes Mellitus (DM), preservation of the left colic artery (ACSP), and the use of a transanal drain (NoCoil), as these were found to be statistically significant in a later chapter. We will delve more into the ACSP factor rather than ICG, given that the effectiveness of ICG has already been thoroughly assessed and studied on data used in this study^1^ and elsewhere^54^. This predictive analysis will be conducted using both classical machine learning methods and a novel quantum-enhanced approach.

Approval for human experiments: This study was approved by the institutional review board of Hospital Nový Jičín a.s., and all experiments were performed in accordance with the relevant guidelines and regulations. Informed consent was obtained from all participants and/or their legal guardians. We have obtained confirmation from the doctors and surgeons who conducted the study that we are permitted to use this data for our research.

This study specifically evaluates the performance of classical and quantum classifiers on raw clinical data without modifications, preserving the natural distribution of cases. This approach ensures ecological validity, providing a realistic assessment of classifier performance in clinical settings. By avoiding techniques like SMOTE or stratified sampling, we maintain the dataset’s inherent imbalance, which reflects the real-world prevalence of anastomotic leak. This allows for a direct and fair comparison between classical and quantum approaches under identical, real-world conditions.

To mitigate overfitting risks, we reduced the number of variables through statistical analysis, including goodness-of-fit tests and risk ratios, and selected features based on medical importance in consultation with clinicians. This feature selection process not only enhances model interpretability but also ensures that the models are trained on clinically relevant predictors, reducing the risk of overfitting despite the small sample size.

Our analysis incorporates several key variables that are critical to understanding the surgical procedure, its immediate impact on the patient, and the patient’s overall health context. These are summarized in Table 1.

**Table 1:** Summary of intraoperative techniques, surgical variables, and patient history factors included in the analysis.

Variable	Description	Values/units
Intraoperative techniques and surgical variables		
NoCoil	Transanal drain used at end of surgery to maintain decompression	Yes/no
ICG	Intraoperative fluorescence angiography (indocyanine green)	Yes/no
ACSP	Preservation of the left colic artery (LCA)	Yes/no
PERFB	Assessment of bowel perfusion using fluorescence angiography	Good/poor
Patient history and contextual variables		
CRP	C-reactive protein, a marker of systemic inflammation	mg/L
HT	Hypertension, characterized by elevated blood pressure	Yes/no
ICHS	Ischemic heart disease	Yes/no
KOAG	Coagulopathy, indicating abnormal blood clotting	Yes/no
DM	Diabetes mellitus, a known risk factor for impaired wound healing	Yes/no
Smoking	Smoking status	Yes/no
Sex	Gender	Male/female
Age	Patient age	Years
BMI	Body mass index	kg/m^2^
HB	Hemoglobin level	g/L
KORT	Corticosteroid therapy (impacts immune response and healing)	Yes/no
ASA	Preoperative physical status classification	2, $>2$
LN	Number of lymph nodes removed	Integer

Among these factors, the preservation of the left colic artery (ACSP variable) plays a crucial role in maintaining blood supply to the descending and sigmoid colon. During colorectal surgery, the preservation of the left colic artery is often considered beneficial for ensuring adequate perfusion (PERFB) of the anastomotic site, thus promoting healing and reducing the risk of anastomotic leak^55^. However, in some cases, its preservation may not be feasible due to anatomical constraints or oncological requirements.

Another factor influencing anastomotic leak risk is the use of a transanal drain. NoCoil refers to a silicone-based tube, typically 1.5 to 2 cm in diameter, inserted into the rectum at the end of the surgery to decompress the rectal stump and protect the anastomosis. This device facilitates passive evacuation of gas and residual stool, reducing intraluminal pressure and helping to minimize the risk of leakage^56^.

Diabetes Mellitus and Smoking have been consistently identified as significant risk factors for anastomotic leak^57^, primarily due to their effects on vascular health and immune response. Similarly, smoking has been shown to impair tissue oxygenation and angiogenesis, further contributing to poor anastomotic healing.

Recent advancements in intraoperative monitoring, such as fluorescence angiography with indocyanine green (ICG), offer a potential method for reducing anastomotic leak incidence by providing real-time visualization of bowel perfusion. Studies indicate that the use of ICG during surgery may improve anastomotic outcomes by allowing surgeons to identify poorly perfused areas and adjust their technique accordingly^58^.

Risk factors such as diabetes, smoking, and the absence of transanal drain have been consistently linked to higher rates of anastomotic leak in surgical literature. A systematic review and meta-analysis by He et al. confirmed that diabetes and smoking significantly increase the risk of anastomotic leak following colorectal surgery, alongside other factors such as male sex and intraoperative blood transfusion^59^. Similarly, Awad et al. highlighted that intraoperative complications, prolonged surgery duration, and inadequate tissue perfusion contribute to higher postoperative anastomotic leak rates^60^.

Novel intraoperative techniques, such as fluorescence angiography (FA), have shown promise in reducing anastomotic leak incidence. Lin et al. conducted a meta-analysis demonstrating that the use of intraoperative indocyanine green (ICG) fluorescence angiography significantly reduces anastomotic leak rates in colorectal resections by improving the assessment of bowel perfusion^61^. More recent work by Balamurugan et al. further supports this, emphasizing that FA leads to better anastomotic outcomes, though additional large-scale studies are needed to validate its routine clinical application^62^.

While existing studies emphasize biological and clinical interventions, recent efforts have explored predictive modeling for anastomotic leak. Venn et al. reviewed preoperative and intraoperative scoring systems designed to predict anastomotic leak occurrence, revealing that although current models provide some predictive power, they remain limited by variability in surgical techniques and patient-specific factors^63^. Our work introduces a computational perspective by leveraging advanced machine learning and quantum computing to enhance anastomotic leak prediction models, potentially improving clinical decision-making and patient outcomes.

### Statistical analysis of patient data

Guided by established clinical literature and surgical constraints, initial exploratory statistical analysis of patient data was performed to assess the significance of the selected explanatory variables. In Table 2, the structure of the patient cohort is presented based on the occurrence of leaks and the treatment methods used. In Table 3, the structure of the patient cohort is presented based on the occurrence of leaks and variables describing the patient’s medical history.

**Table 2:** Structure of patients based on leak occurrence and treatment methods.

Variable	Category	Leak occurred *N* = 28 (14%)	No leak *N* = 172 (86%)	Total *N* = 200	$\widehat{RR}$ (95% CI)	p-value (*χ*^2^ test)
NoCoil	No	25 (17%)	120 (83%)	145	3.16	**0.032**
	Yes	3 (5%)	52 (95%)	55	(0.99; 10.05)	
ICG	No	19 (19%)	81 (81%)	100	2.11	**0.042**
	Yes	9 (9%)	91 (91%)	100	(1.00; 4.44)	
ACSP	No	23 (17%)	112 (83%)	135	2.21	0.074
	Yes	5 (8%)	60 (92%)	65	(0.88; 5.56)	
PERFB	Bad	3 (20%)	12 (80%)	15	1.48	0.757
	Good	25 (14%)	160 (86%)	185	(0.50; 4.34)	

The analysis shows that the occurrence of leaks is significantly associated with the insertion of a special rectal tube (variable NoCoil). As shown in Table 2, the insertion of a special rectal tube reduces the occurrence of leaks by approximately 3.16 times. The 95% confidence interval for the relative risk and the test of independence confirm that this measure has a statistically significant effect on the occurrence of leaks. Another treatment method showing a statistically significant association with leak occurrence is the use of intraoperative fluorescence imaging (ICG), which reduces the occurrence of leaks by 2.11 times. For the other treatment methods studied, no statistically significant effect on leak occurrence was demonstrated, as shown in Table 2.

**Table 3:** Structure of patients based on leak occurrence and patient medical history.

Variable	Category	Leak occurred *N* = 28 (14%)	No leak *N* = 172 (86%)	Total *N* = 200	$\widehat{RR}$ (95% CI)	p-value (*χ*^2^ test)
DM	Yes	9 (25%)	27 (75%)	36	2.16	**0.036**
	No	19 (12%)	145 (88%)	164	(1.06; 4.37)	
Smoking	Yes	9 (26%)	25 (74%)	34	2.31	**0.042**
	No	19 (11%)	147 (89%)	166	(1.15; 4.67)	
HT	Yes	19 (17%)	95 (83%)	114	1.59	0.211
	No	9 (10%)	77 (90%)	86	(0.76; 3.34)	
ASA	$>2$	13 (18%)	59 (82%)	72	1.54	0.215
	2	15 (12%)	113 (88%)	128	(0.78; 3.05)	
CORT	Yes	2 (29%)	5 (71%)	7	2.12	0.564
	No	26 (13%)	167 (87%)	193	(0.62; 7.22)	
Sex	Female	10 (14%)	60 (86%)	70	1.03	0.932
	Male	18 (14%)	112 (86%)	130	(0.50; 2.11)	
COAG	No	27 (14%)	164 (86%)	191	1.27	$>0.999$
	Yes	1 (11%)	8 (89%)	9	(0.19; 8.34)	
ICHS	Yes	3 (14%)	18 (86%)	21	0.98	$>0.999$
	No	25 (14%)	154 (86%)	179	(0.32; 2.96)	

Significant values are in bold.

From Table 3 it is evident that the patient’s medical history most significantly influencing leak occurrence is diabetes mellitus. The presence of diabetes increases the occurrence of leaks by 2.16 times, and the 95% confidence interval for the relative risk, along with the test of independence, confirms that this condition has a statistically significant effect on leak occurrence. Another factor from the patient’s medical history with a statistically significant association with leak occurrence is smoking. Smokers have a 2.31 times higher occurrence of leaks compared to non-smokers. Among smokers and patients with diabetes, leaks occur in approximately 25% of cases. Elevated blood pressure, ASA classification, and corticosteroid use also show notable effects in some patients, as indicated by the relative risk intervals in Table 3, but the influence of these variables on leak occurrence is not statistically significant. For other categorical variables in the patient’s medical history, no statistically significant association with leak occurrence was demonstrated.

For quantitative variables, the description includes the median along with the interquartile range (lower quartile–upper quartile), the point and 95% interval estimate of the difference in medians, and the corresponding Mann-Whitney test, which allows us to determine whether the influence of a given variable is statistically significant.

The description of the quantitative variables related to the patient’s medical history, depending on the occurrence of leaks, the point and 95% interval estimates of median differences (median for patients with a leak—median for patients without a leak), and the corresponding results of the Mann-Whitney test can be found in Table 4.

**Table 4:** Association of quantitative variables with leak occurrence.

Variable	Leak occurred	No leak	Median difference	p-value
CRP	108 (78–131)	62 (34–94)	46 (25; 79)	**<0.001**
BMI	28 (24–31)	26 (24–29)	2 (− 1; 3)	0.348
LN	13 (6–16)	13 (8–17)	0 (− 4; 2)	0.487
Age	65 (60–67)	65 (58–71)	0 (− 4; 2)	0.503
HB	115 (103–121)	116 (107–123)	− 1 (− 7; 4)	0.525

Significant values are in bold.

A statistically significant difference in C-reactive protein (CRP) median levels between patients with and without a leak is observed because a leak represents a type of inflammation, and CRP is a well-established inflammatory marker. As Sproston and Ashworth highlight in *Role of C-reactive protein at sites of inflammation and infection*^64^, CRP’s primary function is its rapid increase in response to inflammatory stimuli. From a medical perspective, CRP should therefore not be considered among potential risk factors that could predict the occurrence of a leak beforehand; instead, it is a consequence marker, reflecting the body’s inflammatory response after a leak has developed.

The timing of the C-reactive protein (CRP) measurement is crucial for its diagnostic value. For none of the other continuous variables was a statistically significant association with leak occurrence observed, see Table 4.

The final predictive model includes NoCoil, ACSP, DM, and Smoking. In clinical practice, when anastomotic leak is suspected, multiple treatment methods, particularly NoCoil and ICG, are often applied together, leading to overlapping effects that reduce ICG’s independent predictive value in the presence of other variables (Table 6). To optimize the model, Akaike’s Information Criterion (AIC) was applied for feature reduction, resulting in model S2 (Table 6), which excluded ICG to improve the AIC score. ACSP, despite its marginal individual significance ($p=0.074$), was retained for its more stable and independent contribution to the model, making it a preferable choice over ICG.

As shown in Table 5, these variables demonstrate strong associations with anastomotic leak ($\chi ^2$ test).

**Table 5:** Summary of selected patient features and their association with anastomotic leak.

Variable	Category	Leak occurred	No Leak	Total	$\widehat{RR}$	p-value
NoCoil	No	25 (17%)	120 (83%)	145	3.16	**0.032**
	Yes	3 (5%)	52 (95%)	55	(0.99; 10.05)	
ACSP	No	23 (17%)	112 (83%)	135	2.21	0.074
	Yes	5 (8%)	60 (92%)	65	(0.88; 5.56)	
DM	Yes	9 (25%)	27 (75%)	36	2.16	**0.036**
	No	19 (12%)	145 (88%)	164	(1.06; 4.37)	
Smoking	Yes	9 (26%)	25 (74%)	34	2.31	**0.042**
	No	19 (11%)	147 (89%)	166	(1.15; 4.67)	

Significant values are in bold.

The figure clearly illustrates the protective effect of NoCoil usage, where patients who received the special rectal tube had anastomotic leak occurrence in only 5% of cases compared to 17% in patients without NoCoil. Similarly, the figure demonstrates the beneficial impact of intraoperative fluorescence imaging (ICG), with leak rates of 9% in the ICG group versus 19% in patients who did not receive this intervention.

Regarding patient risk factors, the figure highlights that diabetes mellitus substantially increases leak risk, with 25% of diabetic patients experiencing anastomotic leak compared to only 12% of non-diabetic patients. The smoking status shows an even more pronounced effect, with smokers having a 26% leak occurrence rate versus 11% in non-smokers. The figure also shows that while ACSP (Acute Care Surgery Program) appears to have a protective trend with 8% leak occurrence compared to 17% without ACSP, this difference did not reach statistical significance ($p=0.074$).

**Fig. 2:**
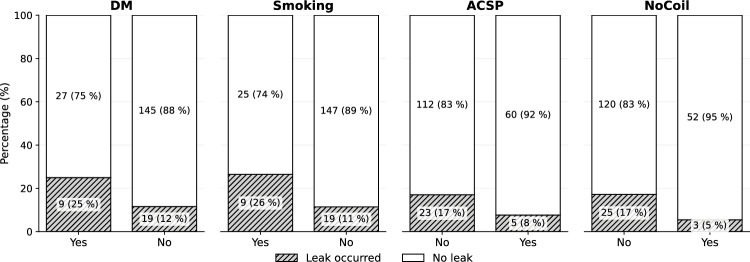
Risk factors of anastomotic leak. To align with standard odds ratio calculations, the category representing the primary risk factor is positioned in the first column. Consequently, ’Yes’ precedes ’No’ for patient comorbidities (DM, smoking), whereas ’No’ precedes ’Yes’ for the application of surgical interventions (ACSP, NoCoil).

These visual comparisons in Fig. 2 effectively demonstrate the substantial clinical impact of both modifiable factors (smoking cessation, NoCoil usage, ICG implementation) and non-modifiable risk factors (diabetes mellitus) on anastomotic leak outcomes, supporting the statistical findings presented in the accompanying tables.

### Multivariate logistic regression models

Building on the initial statistical analysis (Sect. “Statistical analysis of patient data”), which identified significant predictors of anastomotic leak such as NoCoil, ACSP, DM, and Smoking, we now construct multivariate logistic regression models to account for inter-variable relationships. To enhance model interpretability and reduce overfitting, we performed feature reduction from the initial set of 15 variables to focus on the most impactful predictors of anastomotic leak.

In the logistic regression models presented in Table 6, the p-values for each predictor are derived from the Wald test, which evaluates the null hypothesis that a regression coefficient ($\beta$) is zero, indicating no effect on the log-odds of anastomotic leak. The test statistic uses the estimated coefficient and its standard error, with low p-values (e.g., $p < 0.05$) indicating significant predictors.

The comprehensive model (S1) includes all 15 explanatory variables. Unlike individual models, S1 captures interactions between variables, providing a more holistic view of their combined effects on anastomotic leak. To address non-significant variables, we applied Akaike’s Information Criterion (AIC)^65^ for feature reduction, resulting in model S2. This model retains DM, Smoking, NoCoil, and ACSP, which were significant or near-significant in individual analyses. ICG, initially significant in univariate analysis, was excluded due to strong correlations with NoCoil, which reduces its independent predictive power.

Further reduction using the Bayesian Information Criterion (BIC)^66^, which penalizes model complexity more stringently, yields model S3. This model excludes ACSP, retaining DM, Smoking, and NoCoil. The significance of reduced models S2 and S3 was evaluated using likelihood ratio tests ($\chi ^2$ test). Both S2 and S3 are significantly different from the null model ($p < 0.001$ and $p = 0.001$, respectively) but not significantly different from the comprehensive model S1 ($p = 0.879$ and $p = 0.551$, respectively), indicating that they retain full predictive power despite utilizing fewer variables.

Model S4 was constructed using only the variables that were strictly significant at $p < 0.05$ from individual analyses (DM, Smoking, NoCoil, ICG). However, NoCoil and ICG lose significance in S4, likely due to their correlated use in clinical practice, where multiple treatment methods are concurrently applied when anastomotic leak is suspected.

**Table 6:** Evolution of multivariate logistic regression models for anastomotic leak prediction.

Variable	Model S1 (comprehensive)		Model S2 (AIC)		Model S3 (BIC)		Model S4 (Sig. univariate)	
	Coef.	p-value	Coef.	p-value	Coef.	p-value	Coef.	p-value
Constant	6.729	0.061	1.436	0.073	0.319	0.592	0.587	0.361
**DM**	− 0.966	**0.010**	− 1.149	**0.021**	− 1.147	**0.017**	− 1.128	**0.021**
**Smoking**	− 1.380	**0.014**	− 1.429	**0.005**	− 1.261	**0.010**	− 1.376	**0.007**
**NoCoil**	− 1.601	0.055	− 1.610	**0.020**	− 1.439	**0.027**	− 1.051	0.150
**ACSP**	− 0.764	0.249	− 0.952	0.092	–	–	–	–
**ICG**	− 0.577	0.378	–	–	–	–	− 0.633	0.220
Age	− 0.048	0.114	–	–	–	–	–	–
Sex	− 0.391	0.475	–	–	–	–	–	–
BMI	0.014	0.790	–	–	–	–	–	–
ASA	− 0.546	0.348	–	–	–	–	–	–
Hypertension	− 0.484	0.412	–	–	–	–	–	–
ICHS	0.098	0.906	–	–	–	–	–	–
CORT	0.103	0.923	–	–	–	–	–	–
COAG	− 0.746	0.539	–	–	–	–	–	–
Hemoglobin	− 0.006	0.752	–	–	–	–	–	–
PERFB	− 1.283	0.161	–	–	–	–	–	–
Model fit statistics								
Log-likelihood	− 67.0		− 70.0		− 73.0		–	
AIC	166.0		150.0		154.0		–	
LR test vs. Null (*p*)	$<0.001$		$<0.001$		0.001		–	
McFadden’s $R^2$	0.171		0.134		0.098		0.108	
Cox–Snell’s $R^2$	0.129		0.103		0.077		0.084	
Nagelkerke’s $R^2$	0.233		0.185		0.138		0.151	

Significant p-values ($p < 0.05$) are highlighted in bold. AIC: Akaike Information Criterion, BIC: Bayesian Information Criterion.

This step-wise feature reduction mitigates overfitting, enhances interpretability, and focuses on key predictors (DM, Smoking, NoCoil, and potentially ACSP) that drive anastomotic leak risk. By evaluating AIC and BIC criteria alongside pseudo-$R^2$ fit metrics (Table 6), we systematically identified parsimonious models that maintain high predictive power and account for inter-variable relationships which individual univariate models failed to capture. A comprehensive evaluation of the classification performance (e.g., Sensitivity, Specificity, AUC) for these refined classical models is presented alongside the Quantum Neural Network benchmarks in Sect. “Comparative evaluation against classical machinelearning models”.

### Simulating quantum neural networks with noise

To simulate quantum computing behavior as realistically as possible, we implemented a comprehensive noise model that closely approximates the characteristics of real quantum hardware. Our simulation framework utilizes Qiskit’s AerSimulator with a custom noise model that incorporates several key aspects of quantum decoherence and gate errors, providing a realistic testbed for evaluating quantum neural network performance under practical conditions.

The noise model implementation focuses on depolarizing errors, which are among the most significant sources of noise in current quantum hardware. This choice was deliberate as depolarizing noise represents a key paradigm in quantum error modeling that approximates the combined effects of various physical error mechanisms. In actual quantum computers, errors arise from multiple sources including thermal fluctuations, electromagnetic interference, and imperfect control pulses. These varied sources manifest as a combination of bit-flip (*X*), phase-flip (*Z*), and bit-phase-flip (*Y*) errors, which is precisely what the depolarizing channel models.

We selected a gate error probability of $p_{gate} = 0.05$ based on reported error rates from IBM’s Manila quantum processor and similar NISQ devices, where single-qubit gate fidelities typically range from 99.5% to 99.9%, corresponding to error rates of 0.001 to 0.05. Our choice represents a conservative upper bound that accounts for the performance degradation observed in quantum processors under extended operation. This value also aligns with published benchmarks from quantum hardware manufacturers and recent literature on quantum error characterization^67,68^.

We modeled these errors using Kraus operators, which provide a complete description of quantum operations including noise effects. The noise model includes single-qubit gate errors with a probability $p_{gate} = 0.05$, distributed equally among *X*, *Y*, and *Z* errors. The quantum channel effects are represented by Kraus operators with the identity operation (*I*) occurring with probability $1 - p_{gate}$, while Pauli *X*, *Y*, and *Z* operations each occur with probability $p_{gate}/3$. This equal distribution of error types reflects the equiprobable nature of different error mechanisms in depolarizing channels, which is consistent with the quantum information theory principle that noise in quantum systems tends toward maximum entropy in the absence of specific environmental biases. These error channels were applied to all single-qubit gates across all qubits in the system, ensuring comprehensive noise modeling throughout the quantum neural network.

The simulation was configured to use 1024 shots per circuit execution, providing a balance between statistical significance and computational efficiency. This shot count is comparable to typical experimental runs on actual quantum hardware, where 1000-2000 shots are commonly used to overcome the probabilistic nature of quantum measurements while respecting hardware time limitations and queue constraints. Circuit execution was managed through a custom NoiseSimulator class that handles the integration of the noise model with the quantum circuit execution pipeline.

### Data encoding and feature mapping

For encoding classical data into quantum states, we employed the ZZFeatureMap, which is particularly well-suited for machine learning tasks due to its ability to create non-linear feature spaces. The ZZFeatureMap implements a second-order feature map that encodes classical data into quantum states through a series of single-qubit rotations and two-qubit ZZ operations, enabling the quantum neural network to process classical information in a quantum computational framework.

The encoding process begins with an initial rotation layer where each qubit *i* receives a rotation $R_x(x[i])$ based on the input feature *x*[*i*]. This is followed by an entangling layer that applies ZZ operations between pairs of qubits, creating quantum correlations that encode feature interactions. A second rotation layer applies another set of single-qubit rotations, and this process can be repeated for the specified number of repetitions to increase the encoding depth.

Mathematically, the ZZFeatureMap implements the unitary transformation

1 $$\begin{aligned} U(\textbf{x}) = \exp \left( i \sum _{i} x_i Z_i\right) \exp \left( i \sum _{i<j} x_i x_j Z_i Z_j\right) , \end{aligned}$$

where $x_i$ represents the *i*-th feature of the input data, $Z_i$ denotes the Pauli Z operator on qubit *i*, and the second term creates entanglement between qubits based on pairwise products of features. This formulation allows the quantum circuit to capture both linear and quadratic feature combinations, potentially revealing hidden patterns in the data that might not be accessible through classical linear transformations.

The feature mapping circuit for 4 features with single repetition exhibits a depth of 17 with a total of 26 individual quantum gates, comprising 12 CNOT gates for entanglement generation, 10 parameterized phase gates for feature encoding, and 4 Hadamard gates for superposition initialization. This gate composition reflects the circuit’s emphasis on creating quantum correlations through the CNOT operations while encoding classical feature information through the parameterized phase rotations. The relatively moderate depth of 17 time steps ensures that the feature encoding process remains feasible on near-term quantum devices while still providing sufficient complexity to create meaningful quantum feature spaces for the 4-dimensional input data. Circuit depth represents a critical performance metric that directly impacts practical implementation, as it refers to the number of sequential time steps required for execution, with lower depth generally implying shorter execution time and reduced exposure to decoherence effects that can degrade quantum states over time.

**Fig. 3:**
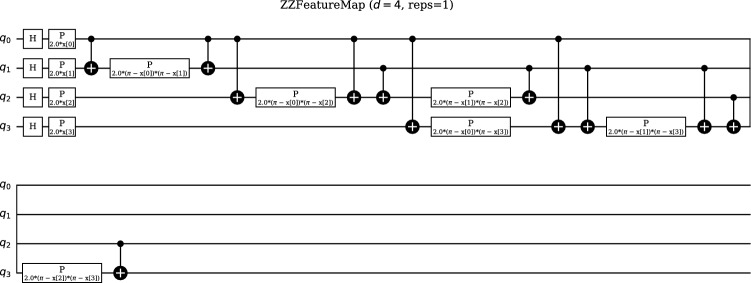
The ZZFeatureMap applies Hadamard gates to initialize superposition, followed by entangling CNOT gates and parameterized phase gates $P(2.0\phi (x_i, x_j))$ to encode feature interactions. This combination enables quantum interference, where correlations between input data are mapped into the entangled quantum state.

In our implementation, we configured the ZZFeatureMap with 4 feature dimensions and a single repetition of the encoding circuit. This configuration maintains the dimensionality of the input space while creating non-linear feature combinations through quantum operations. The quantum circuit representing the ZZFeatureMap is shown in Fig. 3, which illustrates how Hadamard gates initialize superposition, followed by entangling CNOT gates and parameterized phase gates $P(2.0\phi (x_i, x_j))$ to encode feature interactions. This combination enables quantum interference, where correlations between input data are mapped into the entangled quantum state, providing the foundation for the subsequent variational quantum processing.

For instance, Havlíček et al.^14^ demonstrated the potential of quantum-enhanced feature spaces, prominently featuring the ZZFeatureMap, to make classically challenging datasets amenable to linear classification. Furthermore, its empirical effectiveness has been observed in various studies utilizing quantum kernel methods and Variational Quantum Classifiers^69^.

### Variational quantum circuit design

Following the data encoding stage, the quantum neural network employs variational ansatze to process the encoded information through parameterized quantum circuits. The selection of appropriate ansatze involves balancing expressive power with computational efficiency, as more complex circuits can represent richer quantum states but require increased computational resources and may be more susceptible to noise effects. We investigated two prominent ansatze that offer different trade-offs between these competing requirements: the RealAmplitudes ansatz and the EfficientSU2 ansatz.

**Fig. 4:**
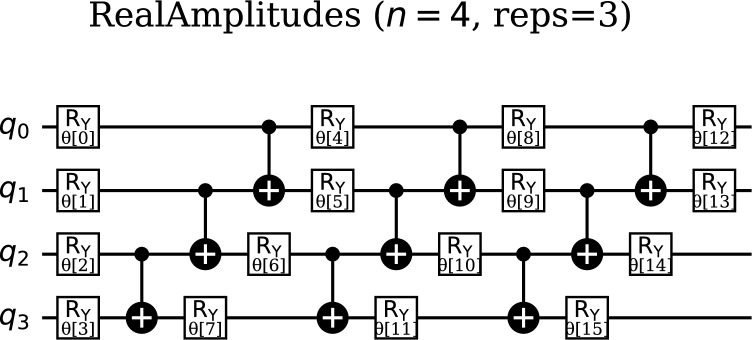
Quantum circuit diagram of the RealAmplitudes ansatz, featuring a layered structure with alternating single-qubit $R_y$ rotations and multi-qubit entangling gates (e.g., CNOT). This ansatz generates quantum states with RealAmplitudes, simplifying interpretation while maintaining expressiveness for complex data. Its gate efficiency reduces computational costs, making it a practical choice for variational quantum circuits.

The RealAmplitudes ansatz represents a well-established choice for variational quantum classifiers due to its simplicity and computational efficiency. This ansatz utilizes a layered structure consisting of alternating single-qubit rotations ($R_y$ gates) and multi-qubit entangling gates (typically CNOT gates), as illustrated in Fig. 4. The layered architecture allows for systematic construction of expressive quantum states while maintaining a relatively shallow circuit depth, which is crucial for maintaining coherence in noisy quantum environments.

The RealAmplitudes ansatz with 4 qubits and 3 repetitions demonstrates favorable circuit characteristics with a depth of 11 and a total of 25 quantum gates, reflecting its design philosophy of computational efficiency while maintaining expressive capability. The gate composition emphasizes rotation operations for parameterization while utilizing entangling gates judiciously to create necessary quantum correlations. This relatively shallow depth of 11 time steps, achieved through the efficient layering of the 3 repetitions, minimizes the exposure to decoherence effects, making it particularly suitable for near-term quantum implementations where coherence times are limited. When combined with the ZZFeatureMap, the complete RealAmplitudes quantum neural network achieves a total circuit depth of 28 with 51 gates, representing an efficient balance between expressiveness and practical implementability on current quantum hardware.

A defining characteristic of the RealAmplitudes ansatz is its generation of quantum states with only RealAmplitudes, which simplifies the interpretation and analysis of the circuit’s output since all basis state probabilities are represented by real numbers. This property also reduces the parameter space that must be explored during optimization, potentially leading to more efficient training procedures. Additionally, the RealAmplitudes ansatz requires a relatively low number of gates compared to more intricate ansatze, translating to lower computational costs for circuit implementation and reduced susceptibility to accumulated gate errors in noisy environments.

However, the simplicity that makes the RealAmplitudes ansatz computationally attractive also imposes limitations on its expressive capabilities. Compared to more complex ansatze, it may have reduced ability to represent highly intricate quantum states, which could potentially limit its performance for tasks demanding the most sophisticated quantum computations. Furthermore, the restriction to RealAmplitudes may prevent the circuit from fully exploiting quantum interference effects that require complex phases.

**Fig. 5:**
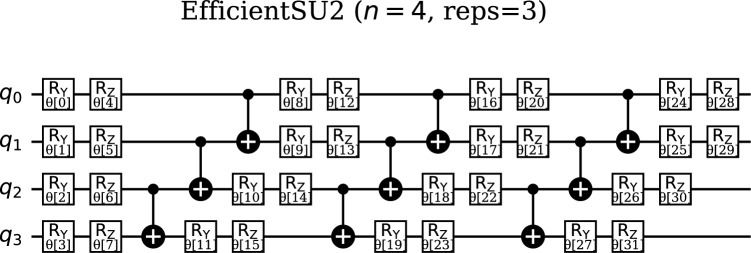
Quantum circuit diagram of the EfficientSU2 ansatz used in the quantum neural networks. The circuit features a layered structure with parametrized single-qubit rotations around two axes (typically $R_x$ and $R_y$), followed by entangling gates to create correlations between qubits.

In contrast, the EfficientSU2 ansatz offers enhanced flexibility through a richer parameterization scheme. This ansatz retains the beneficial layered structure but utilizes single-qubit rotations around two axes (typically $R_x$ and $R_y$) followed by entangling gates, as shown in Fig. 5. The additional rotation axis provides access to a broader range of single-qubit unitaries, enabling the circuit to explore a more extensive portion of the quantum state space and potentially capture more complex data patterns.

The EfficientSU2 ansatz with 4 qubits and 3 repetitions exhibits increased circuit complexity with a depth of 15 and a total of 41 quantum gates, reflecting its enhanced expressiveness through additional parameterized rotations across multiple axes. The gate distribution includes rotations around both x and y axes within each repetition layer, providing greater flexibility in quantum state preparation and potentially enabling more sophisticated pattern recognition capabilities. However, this increased depth of 15 time steps compared to the RealAmplitudes 11 steps represents a trade-off between expressiveness and susceptibility to noise, requiring careful consideration of the coherence properties of the target quantum hardware. The complete EfficientSU2-based quantum neural network, when combined with the ZZFeatureMap, results in a total circuit depth of 32 with 67 gates, demonstrating the computational overhead associated with enhanced quantum expressiveness.

The increased expressive power of the EfficientSU2 ansatz stems from its ability to generate quantum states with complex amplitudes and phases, allowing for more sophisticated quantum interference patterns. This enhanced expressiveness can translate to superior performance for certain classification tasks, particularly those involving complex, non-linearly separable data structures. The additional degrees of freedom provided by the dual-axis rotations enable the ansatz to adapt more flexibly to diverse data characteristics during the optimization process.

However, this enhanced capability comes with computational trade-offs. The EfficientSU2 ansatz typically requires more gates than the RealAmplitudes ansatz, leading to increased circuit depth and higher computational burden for both circuit implementation and optimization. The larger parameter space also presents optimization challenges, as the increased dimensionality can lead to more complex loss landscapes with additional local minima, potentially making the training process more difficult and requiring more sophisticated optimization strategies.

The choice between these ansatze ultimately depends on the specific requirements of the machine learning task, the available computational resources, and the noise characteristics of the target quantum hardware. In our comparative study, both ansatze were evaluated under identical noisy conditions to assess their relative performance and robustness, providing insights into the practical trade-offs between circuit complexity and classification accuracy in realistic quantum computing environments.

### Variational parameters optimization

To optimize the parameters of our quantum neural networks, we employed a diverse set of optimization algorithms, each offering distinct advantages for navigating the complex parameter landscapes inherent in variational quantum circuits. The selection encompasses both gradient-based and gradient-free methods, providing robustness against the challenges posed by noisy quantum environments and barren plateaus (Table 7).

**Table 7:** Classification of optimizers by principle.

Optimizer	Category
BFGS	Gradient-based method
SLSQP	Gradient-based method
CMA-ES	Metaheuristic method
COBYLA	Gradient-free method
SPSA	Gradient-free method

BFGS is a quasi-Newton optimization method that approximates the inverse Hessian matrix using gradient information to achieve rapid convergence for smooth objective functions^70^. In quantum circuit optimization, BFGS can effectively navigate parameter spaces when gradients are accessible, though its performance may degrade in the presence of sampling noise and measurement uncertainties typical in quantum hardware.

CMA-ES represents a derivative-free evolutionary strategy that maintains a population of candidate solutions while adapting a multivariate normal distribution to guide the search process^71^. This metaheuristic approach proves particularly valuable for quantum optimization as it naturally handles the non-convex, multimodal landscapes often encountered in variational circuits and remains robust to noise inherent in quantum measurements^72–74^.

COBYLA employs a simplex-based approach to construct linear approximations of the objective function, making it well-suited for noisy optimization environments^75^. Its derivative-free nature allows it to handle the stochastic fluctuations common in quantum circuit evaluation, though convergence may be slower compared to gradient-based methods in noiseless scenarios.

SLSQP combines sequential quadratic programming with constraint handling capabilities, constructing quadratic approximations to iteratively refine solutions^76^. While effective for smooth, differentiable problems, its reliance on gradient information can be challenging in quantum settings where parameter shift rules or finite differences must be employed for gradient estimation.

SPSA offers a highly efficient gradient-free approach that estimates gradients using only two function evaluations per iteration, regardless of parameter dimensionality^77^. This efficiency makes it particularly attractive for quantum optimization where circuit evaluations are computationally expensive, and its inherent robustness to noise aligns well with the stochastic nature of quantum measurements and decoherence effects.

The optimization algorithms considered have the following hyperparameters and initialization settings, assuming $m$ parameters where applicable: CMA-ES uses an initial standard deviation $\sigma _0 = 0.15$, a population size of $\lceil 4 + 3 \log (m) \rceil$, a parent fraction $\mu = 0.5$, a mean update factor $c_{\text {mean}} = 1.0$, and a damping factor of 1.0; SPSA is configured with a maximum of 75 iterations , an allowed increase of $10^{-3}$, blocking enabled, and a termination checker with $N=10$; BFGS, specifically L-BFGS-B, is initialized with a maximum of 75 iterations and a function tolerance of $10^{-4}$; SLSQP is set with a maximum of 75 iterations and a function tolerance of $10^{-4}$; COBYLA is configured with a maximum of 75 iterations.

### Predictive modeling approaches

Our study compares classical and quantum-enhanced approaches to predictive modeling of anastomotic leak:

1. *Classical methods:* We employed a diverse set of classical machine learning models, including logistic regression (LR), linear discriminant analysis (LDA), Support Vector Machines (SVM), Multi-Layer Perceptron (MLP)^78^, and Nearest Neighbors (NN). Additionally, we utilized ensemble methods such as Gradient Boosting Machines (GBM)^79^ and AdaBoost^80^ to improve predictive performance. The implementation of these classical models was carried out using the scipy^81^ module in Python. The effectiveness of these models was evaluated using Receiver Operating Characteristic (ROC) curves, with a primary focus on sensitivity (true positive rate) to prioritize identification of patients at high risk of anastomotic leak.
2. *Quantum neural networks:* A novel quantum-enhanced approach using Variational Quantum Circuits^82^ is applied to the dataset. These circuits leverage the principles of quantum mechanics to explore high-dimensional feature spaces, potentially improving the predictive power for imbalanced datasets. We used the qiskit library^83^ for all the work with quantum circuits.

Model performance is evaluated using the following metrics:

- *ROC curves and AUC:* The Area Under the Curve (AUC) of the Receiver Operating Characteristic (ROC) curve measures a model’s ability to discriminate between patients with and without the complication. AUC values closer to 1 indicate better performance, as the model correctly distinguishes between positive and negative cases more often.
- *Sensitivity and specificity:* Sensitivity (true positive rate) reflects the model’s ability to correctly identify patients who experience the complication, ensuring high-risk individuals are not missed. Specificity (true negative rate) measures how well the model correctly identifies those who do not develop the complication, reducing false positives.
- *Predictive power, calibration, and classification accuracy:* We assess overall model performance using various statistical measures:
  - *Efron’s R*^2^*, McKelvey & Zavoina R*^2^*, and Count R*^2^ evaluate how well the model explains variance in the outcome.
  - *Brier Score and Log Loss* assess model calibration, measuring how well predicted probabilities align with actual outcomes.
  - *F1 Score* balances precision and recall, particularly relevant for imbalanced datasets.

Additionally, Positive Predictive Value (PPV) and Negative Predictive Value (NPV) provide practical insights into prediction reliability. PPV represents the probability that a positive prediction corresponds to an actual occurrence of the complication, while NPV indicates the probability that a negative prediction correctly identifies a complication-free patient. These metrics are particularly useful in clinical decision-making, where understanding the likelihood of correct predictions is crucial.

To rigorously evaluate and compare the performance of our predictive models, we established a comprehensive evaluation framework. This framework encompasses cross-validation to assess robustness, ROC curve analysis for threshold optimization, and a comparative analysis of classification metrics at fixed sensitivity levels. These methodologies are designed to provide a multifaceted understanding of each model’s strengths and limitations in the context of rare post-surgical complication prediction.

To ensure the reliability and generalizability of our findings, we employed cross-validation (CV). This technique is crucial for evaluating how well models generalize to unseen data and for mitigating the risk of overfitting. By systematically training and testing models on different partitions of the dataset, cross-validation provides a more robust estimate of predictive performance than a single train-test split.

### Feature importance analysis for quantum neural network

Interpreting the predictive mechanisms of machine learning models is paramount for their application in critical domains such as clinical risk assessment. While classical linear models like logistic regression offer straightforward interpretability through their model coefficients, the complex, high-dimensional nature of QNNs presents a significant challenge. In our work, the association between clinical risk factors DM, Smoking, ACSP, and NoCoil and post-surgical anastomotic leak was modeled using a QNN in Qiskit. This QNN was constructed with a ZZFeatureMap to encode the four-dimensional feature vector into an entangled quantum state, and an EfficientSU2 ansatz to create a trainable parameterized quantum circuit. Optimization was performed via a CMA-ES.

Unlike a logistic regression model, where the feature importance is directly quantified by the coefficients $\beta _i$ in the equation

2 $$\begin{aligned} \ln \left( \frac{p}{1-p}\right) = \beta _0 + \sum _i \beta _i x_i, \end{aligned}$$

the trained parameters of a QNN lack a direct, human-interpretable meaning. Consequently, to understand our model’s decision-making process, we explored two experimental, perturbation-based feature importance techniques. These methods aim to approximate a feature’s influence by observing the model’s behavior when its inputs are systematically altered.

Our first experimental approach was a permutation-based analysis, a model-agnostic technique that assesses the global impact of a feature on the model’s overall performance. This method first establishes a baseline predictive accuracy, measured by the AUC on the test set. Then, for each feature, its values are randomly shuffled across all samples, effectively breaking any learned relationship between that feature and the outcome. The model’s AUC is re-evaluated on this perturbed data, a process repeated one hundred times for statistical robustness. The average decrease in AUC is then taken as the importance score for that feature, with a larger drop indicating a greater reliance of the model on that variable. While this method captures complex, non-linear interactions as reflected in the final performance metric, its stochastic nature can lead to variance in the results, and the shuffling process may create unrealistic data instances that disrupt the very feature correlations the ZZFeatureMap is designed to learn.

To address these limitations, we implemented a second experimental technique: a gradient-based importance analysis. This method provides a more direct and stable probe of the model’s sensitivity to each feature. Instead of the large-scale disruption of shuffling, it applies a minute, controlled perturbation ($\epsilon$) to a single feature at a time and measures the immediate impact on the model’s output probability. The importance is quantified as the average absolute change in the predicted probability across the entire test set. This value serves as a numerical approximation of the gradient of the model’s output with respect to its input, effectively measuring how much the model’s prediction changes for a small change in a given feature. This approach is deterministic and better preserves the intricate, learned correlations between features, offering a more nuanced view of a feature’s local influence on the classification decision.

In contrasting these approaches, the permutation method provides a holistic score of a feature’s contribution to the final AUC, whereas the gradient method reveals the model’s internal sensitivity to variations in the feature’s value. Both quantum-inspired techniques stand apart from logistic regression by their ability to account for the non-linear relationships and entanglement-based correlations captured by the QNN. While the classical approach provides a clear, linear, but potentially incomplete picture, our experimental methods represent a necessary step toward unpacking the “black box” of quantum classifiers, allowing us to build confidence in their predictions and gain deeper insights from the complex patterns they uncover. The development of such interpretability tools is a vital research direction for the advancement and practical deployment of quantum machine learning in scientific discovery and beyond.

## Results

Building on the statistical insights from Sect. “Statistical analysis of patient data”, where NoCoil, ACSP, DM, and Smoking emerged as key predictors of anastomotic leak, we now evaluate the performance of QNNs for this clinical classification task. The analysis begins by optimizing variational parameters in quantum circuits under realistic noise conditions, comparing the convergence and stability of ansatze like RealAmplitudes (RA) and EfficientSU2 (ESU2) when paired with classical optimizers such as BFGS and CMA-ES. These optimized QNNs are then benchmarked against classical machine learning models, including Logistic Regression and Multi-Layer Perceptrons, using metrics tailored to clinical needs, such as sensitivity-driven thresholds and probability calibration.

### Optimization of variational parameters in quantum neural network ansatze

This subsection focuses on the mean convergence plots of various optimization algorithms when applied to both the RA and ESU2 ansatze in a quantum neural network. The results presented here are averaged over 10 independent runs for each optimizer-ansatz pair. It is crucial to note that this optimization was performed in a noisy environment, incorporating sampling noise (1024 shots) and realistic modeled errors, including quantum decoherence and gate errors, to simulate practical quantum computing conditions. Individual convergence traces for each run can be found in Appendix A.

**Fig. 6:**
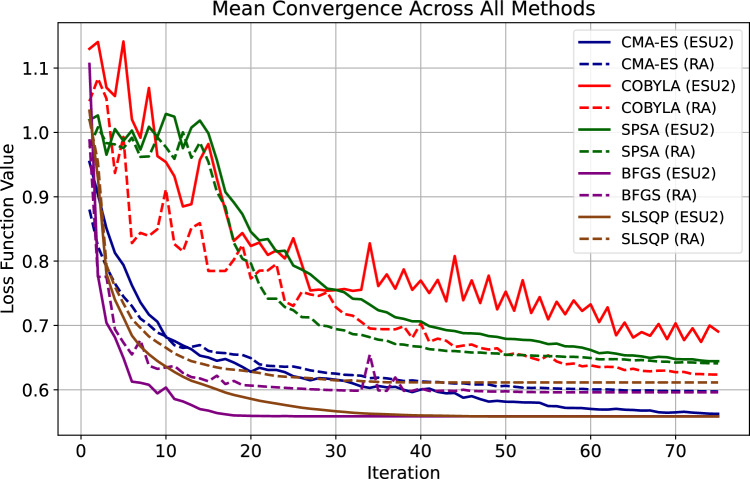
Mean convergence of loss function values over 10 runs for RealAmplitudes (RA) and EfficientSU2 (ESU2) ansatze, optimized using different algorithms. The optimization includes sampling noise (1024 shots) and modeled quantum decoherence and gate errors.

The mean convergence plots, shown in Fig. 6, illustrate the performance of different optimizers in minimizing the loss function. The summarized results for the mean final error and their standard deviations over the 10 runs are presented in Table 8.

**Table 8:** Summarized mean final error and standard deviation for different optimizers and ansatze (Mean over 10 Runs).

Optimizer	Ansatz	Mean error	Std Dev
BFGS	ESU2	0.559	0.009
BFGS	RA	0.596	0.010
CMA-ES	ESU2	0.563	0.010
CMA-ES	RA	0.597	0.012
COBYLA	ESU2	0.691	0.029
COBYLA	RA	0.624	0.016
SLSQP	ESU2	0.559	0.011
SLSQP	RA	0.611	0.018
SPSA	ESU2	0.645	0.036
SPSA	RA	0.641	0.027

As observed from Table 8, the SLSQP optimizer achieved the lowest mean final error for the ESU2 ansatz ($0.559 \pm 0.011$), followed closely by BFGS with ESU2 ($0.559 \pm 0.009$) and CMA-ES with ESU2 ($0.563 \pm 0.010$). These three optimizers consistently demonstrated superior performance across both ansatze, converging to lower loss function values with relatively small standard deviations. This indicates robust optimization capabilities even within a noisy quantum environment characterized by sampling noise (1024 shots) and modeled hardware errors, such as decoherence and gate inaccuracies.

Comparing the two ansatze, ESU2 generally yielded lower mean final errors when paired with top-performing optimizers. For instance, the lowest overall error was achieved by SLSQP with ESU2. Conversely, COBYLA exhibited higher final errors, particularly with the ESU2 ansatz ($0.691 \pm 0.029$), though it performed marginally better with the RA ansatz ($0.624 \pm 0.016$). SPSA demonstrated the weakest performance among the tested optimizers, characterized by higher mean final errors (e.g., $0.645 \pm 0.036$ for ESU2) and larger standard deviations, suggesting less stable convergence in the presence of noise. This analysis underscores the necessity of selecting resilient optimizers for training quantum neural networks under realistic noisy conditions.

### QNN performance analysis across multiple metrics

**Fig. 7:**
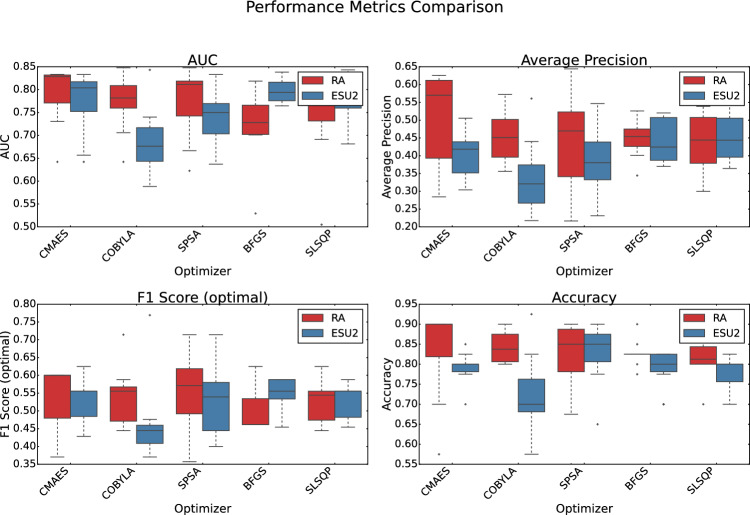
Comparison of QNN models (RealAmplitudes vs. EfficientSU2 ansatze) across different optimizers, evaluated by standard performance metrics (AUC, Average Precision, F1 Score, and Accuracy). Box plots show the distribution of metrics over 10 independent runs.

**Fig. 8:**
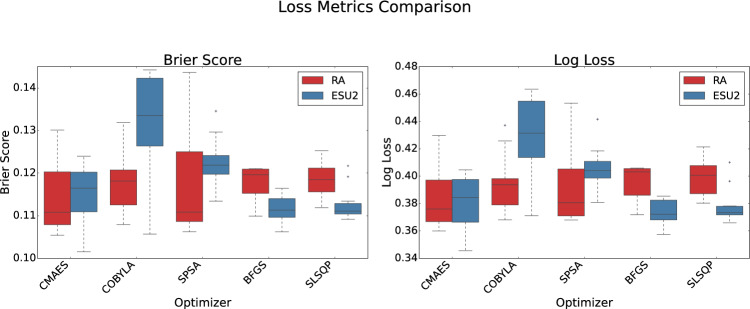
Comparison of QNN models (RealAmplitudes vs. EfficientSU2 ansatze) across different optimizers, measured by loss metrics (Brier Score and Log Loss). Box plots show the distribution of metrics over 10 independent runs.

**Fig. 9:**
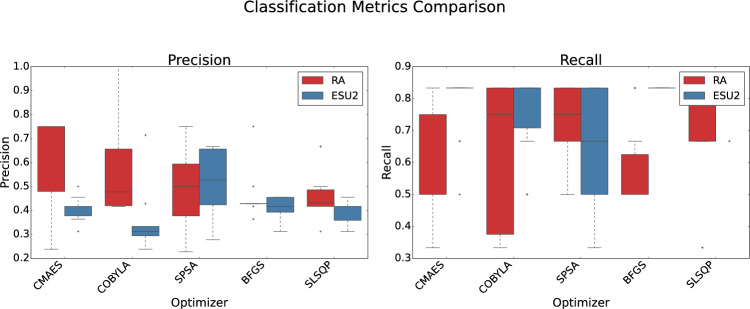
Comparison of QNN models (RealAmplitudes vs. EfficientSU2 ansatze) across different optimizers, evaluated by classification metrics (Precision and Recall). Box plots show the distribution of metrics over 10 independent runs.

**Fig. 10:**
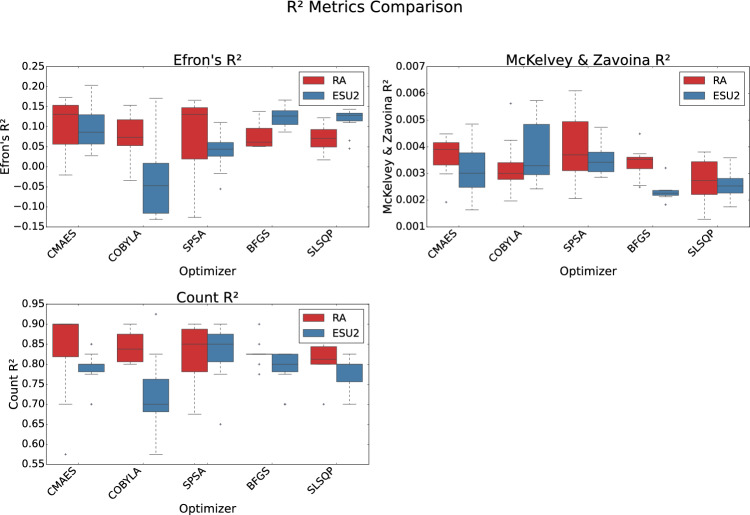
Comparison of QNN models (RealAmplitudes vs. EfficientSU2 ansatze) across different optimizers, evaluated by pseudo-$R^2$ scores (Efron’s, McKelvey & Zavoina’s, and Count). Box plots show the distribution of metrics over 10 independent runs.

We evaluated two ansatze: RA and ESU2, paired with six optimization algorithms: Broyden-Fletcher–Goldfarb–Shanno (BFGS), CMA-ES, Constrained Optimization by Linear Approximation (COBYLA), Simultaneous Perturbation Stochastic Approximation (SPSA), Sequential Least Squares Programming (SLSQP), and one additional optimizer. To account for the stochastic nature of quantum circuit sampling and parameter training, we conducted 10 independent runs for each optimizer-ansatz pair, ensuring statistical robustness. Performance, loss, classification, and pseudo-$R^2$ metrics are visualized in Figs. 7, 8, 9 and 10, using box plots to illustrate metric distributions over these runs.

The overall performance of QNN models is summarized in Fig. 7, which displays the Area Under the Receiver Operating Characteristic Curve, Average Precision, F1 Score, and Accuracy. The ESU2-BFGS combination achieved the highest mean AUC of $0.797 \pm 0.024$, indicating strong discriminative power in distinguishing patients with and without post-surgical complications. For Average Precision, critical for imbalanced medical datasets, the RA-CMA-ES model excelled with a score of $0.504 \pm 0.121$. The RA-SPSA configuration provided the best F1 Score ($0.557 \pm 0.103$), balancing precision and recall effectively. The highest accuracy was achieved by RA-COBYLA ($0.845 \pm 0.038$), though accuracy alone can be misleading in imbalanced datasets. These results highlight that different ansatz-optimizer pairs excel in specific performance aspects, underscoring the importance of task-specific configuration selection.

Figure 8 presents the Brier Score and Log Loss, which assess predictive accuracy and probability calibration. Lower values indicate better-calibrated predictions. The ESU2-BFGS model achieved the lowest Brier Score ($0.112 \pm 0.003$) and Log Loss ($0.374 \pm 0.009$), with small standard deviations indicating stable convergence and reliable probability estimates. In contrast, gradient-free optimizers like CMA-ES and COBYLA exhibited higher variance in loss metrics, reflecting their susceptibility to the stochasticity of quantum optimization landscapes.

The precision-recall trade-off, crucial for medical applications, is illustrated in Fig. 9. The RA-CMA-ES model achieved the highest mean precision ($0.613 \pm 0.196$), making it suitable for minimizing false positives, a priority in scenarios where misclassification carries significant consequences. Conversely, the ESU2-BFGS model demonstrated perfect and consistent recall ($0.833 \pm 0.001$), ensuring all positive cases were identified, a critical feature for medical diagnostics where missing complications can be detrimental. The RA ansatz generally showed higher precision but with greater variance, while ESU2 maintained consistency in recall.

The goodness-of-fit of the models is evaluated using pseudo-$R^2$ metrics, shown in Fig. 10. The ESU2-BFGS model achieved the highest Efron’s $R^2$ ($0.125 \pm 0.0230$), indicating a better fit to the data distribution. The RA-COBYLA model led in Count $R^2$, consistent with its high accuracy. However, McKelvey and Zavoina’s $R^2$ values were low across all models, with RA-SPSA performing best ($0.004 \pm 0.001$), suggesting limited explained variance, typical for complex medical classification tasks with inherent noise.

**Table:** 

*Connection to Variational Parameter Optimization:* The effective optimization of variational parameters in the parameterized quantum gates of the ansatze directly influences all downstream performance metrics. The superior convergence achieved by optimizers like BFGS, CMA-ES, and SLSQP with the ESU2 ansatz (as shown in Table 8) translates into better calibrated probabilities, improved classification accuracy, and more reliable clinical predictions	

The comprehensive QNN analysis reveals the complex interplay between ansatz and optimizer in quantum neural network performance. The ESU2-BFGS combination consistently excelled in AUC, loss metrics, and recall, making it ideal for applications requiring reliable class separation and probability calibration. The RA ansatz, particularly with CMA-ES, demonstrated superior precision and robustness, as evidenced by smaller interquartile ranges and fewer outliers in box plots, making it suitable for minimizing false positives. CMA-ES’s evolutionary approach proved particularly robust against the stochasticity of quantum optimization, including shot noise and gate errors, offering stable performance across runs. This reliability is critical for practical deployment in medical settings.

No single configuration dominated across all metrics, highlighting the need for task-specific optimization. For instance, ESU2-BFGS is preferable for high recall and calibration, while RA-CMA-ES suits precision-critical tasks. The observed performance variations emphasize the necessity of comprehensive empirical evaluation in quantum machine learning, particularly for medical classification, where both accuracy and reliability are paramount.

A key finding of our analysis is the superior probability calibration of classical models like MLP compared to the QNNs. This weaker calibration in QNNs may stem from several factors inherent to the variational quantum approach: the stochasticity of shot-noise in the measurement process, the complexity of the optimization landscapes which can lead to convergence in local minima, and the impact of simulated hardware noise which can impair the fine-tuning of output probabilities. While QNNs excelled at the classification task of separating high-risk from low-risk patients, their raw output probabilities are less reliable. Future work should investigate post-hoc calibration techniques, such as Platt scaling or isotonic regression, which could be applied to QNN outputs to improve their reliability without sacrificing their classification strength. Developing hybrid quantum-classical training methodologies that explicitly optimize for a calibration-aware loss function is another promising research direction.

### Comparative evaluation against classical machine learning models

To evaluate the performance of QNNs against established classical machine learning models for predicting rare post-surgical complications, specifically Anastomotic leak, we conducted a comprehensive assessment using multiple performance metrics. The classical models: Logistic Regression, AdaBoost, Linear Discriminant Analysis, Gaussian Naive Bayes, and Multi-Layer Perceptron, were optimized with fine-tuned hyperparameters to utilize their full potential (hyperparameters described in detail in Section D). This ensures a fair comparison by leveraging the optimal configuration of each classical approach.

Anastomotic leak, occurring in approximately 14% of patients, is a severe complication where high sensitivity (recall) is critical to ensure timely identification and intervention. To enable a fair comparison, we optimized the decision threshold for each model to achieve the highest possible sensitivity while using an F-beta score to balance precision and recall, prioritizing the detection of true positives. This approach reflects the clinical importance of minimizing false negatives, as missing an anastomotic leak can lead to life-threatening consequences.

For QNNs, which incorporate variational quantum circuits with RA or ESU2 ansatze and are optimized using CMA-ES, COBYLA, SPSA, BFGS, or SLSQP, the reported metrics represent the mean performance over 10 runs due to their stochastic nature. In contrast, deterministic classical models were evaluated based on a single optimized run, while non-deterministic classical models (e.g., MLP, Random Forest) were averaged over multiple runs to ensure a fair comparison with quantum stochasticity.

Table 9 presents a detailed comparison of model performance at a fixed sensitivity of 83%, achieved by adjusting thresholds to maximize sensitivity while optimizing the F-beta score. The table includes both threshold-dependent metrics (Count R^2^, F1 Score, Specificity, PPV, NPV, Accuracy) and probability calibration metrics (Efron’s R^2^, McKelvey & Zavoina R^2^, Brier Score, Log Loss).

Among the classical models, MLP demonstrates the highest Efron’s R^2^ (0.191) and the lowest Brier Score (0.103) and Log Loss (0.359), indicating strong calibration and discriminative power, though its F1 Score (21%) is the lowest due to lower precision. GNB achieves the highest Count R^2^ (0.500) and F1 Score (33%) among classical models, with a Specificity of 44% and PPV of 21%, reflecting a balanced performance. LR, LDA, and AdaBoost show moderate performance, with Count R^2^ values ranging from 0.400 to 0.450 and F1 Scores from 29% to 31%.

QNNs generally outperform classical models across most metrics. QNN - COBYLA - RA achieves the highest Count R^2^ (0.845) and Accuracy (85%), with a strong F1 Score (54%) and Specificity (58%). QNN - BFGS - ESU2 shows the highest Efron’s R^2^ (0.125) among QNNs and a competitive Brier Score (0.112) and Log Loss (0.374), alongside a high Specificity (66%) and PPV (32%). QNN - CMA-ES - RA and QNN - SPSA - ESU2 also perform well, with Count R^2^ values of 0.835 and 0.830, respectively, and F1 Scores of 54% and 53%. Notably, QNN - CMA-ES - RA achieves the highest NPV (96%), indicating excellent performance in ruling out non-cases. However, QNN - COBYLA - ESU2 underperforms, with a negative Efron’s R^2^ (− 0.034) and the highest Brier Score (0.132) and Log Loss (0.430), suggesting poorer calibration for this configuration.

**Table 9:** Performance comparison of predictive models at a fixed sensitivity of 83% (thresholds optimized via F-beta score). QNN metrics represent the mean across 10 independent runs; classical models represent a single optimized run.

Model	Threshold	Classification metrics					Probability calibration & fit			
		Acc.	Spec.	PPV	NPV	F1	Brier	Log Loss	Efron’s $R^2$	Count $R^2$
Classical machine learning models										
Logistic Regression (LR)	0.156	40%	32%	18%	92%	29%	0.118	0.390	0.072	0.400
AdaBoost	0.280	45%	38%	19%	93%	31%	0.134	0.443	− 0.054	0.450
LDA	0.135	40%	32%	18%	92%	29%	0.115	0.381	0.095	0.400
Gaussian Naive Bayes	0.410	50%	44%	21%	94%	33%	0.116	0.393	0.087	0.500
Multi-Layer Perceptron	0.133	45%	38%	19%	93%	21%	0.103	0.359	0.191	0.425
Quantum neural networks (QNN)										
QNN - CMA-ES - RA	0.290	84%	64%	29%	96%	54%	0.114	0.384	0.103	0.835
QNN - CMA-ES - ESU2	0.280	79%	58%	29%	85%	53%	0.115	0.381	0.099	0.793
QNN - COBYLA - RA	0.260	85%	58%	26%	95%	54%	0.119	0.395	0.070	0.845
QNN - COBYLA - ESU2	0.260	73%	49%	23%	93%	47%	0.132	0.430	− 0.034	0.728
QNN - SPSA - RA	0.230	81%	58%	26%	95%	56%	0.117	0.392	0.082	0.805
QNN - SPSA - ESU2	0.240	83%	53%	24%	95%	53%	0.122	0.406	0.040	0.830
QNN - BFGS - RA	0.210	83%	35%	21%	60%	50%	0.118	0.395	0.077	0.828
QNN - BFGS - ESU2	0.320	79%	66%	32%	96%	55%	0.112	0.374	0.125	0.788
QNN - SLSQP - RA	0.220	82%	47%	22%	90%	53%	0.119	0.399	0.071	0.815
QNN - SLSQP - ESU2	0.340	78%	65%	34%	86%	53%	0.113	0.379	0.115	0.780

Acc: Accuracy, Spec: Specificity.

The results highlight the potential of QNNs, particularly with RA and ESU2 ansatze optimized by COBYLA, CMA-ES, or BFGS, to outperform classical models in detecting anastomotic leak at high sensitivity. The superior Count R^2^, Accuracy, and NPV of QNNs suggest they are effective at both identifying true positives and minimizing false positives, critical for clinical applications. However, the variability in QNN performance across optimizers and ansatze underscores the importance of careful configuration selection.

The results in Table 9, evaluated at a fixed sensitivity of 83% with F-beta optimized thresholds to prioritize anastomotic leak detection, reveal distinct performance profiles for QNNs and classical models. QNNs generally show stronger threshold-dependent metrics (Count R^2^, F1 Score, Specificity, PPV, NPV, Accuracy), particularly with certain ansatz-optimizer combinations, making them effective for identifying true positives and minimizing false positives in clinical screening. However, their probability calibration metrics (Efron’s R^2^, Brier Score, Log Loss) vary, with some configurations exhibiting weaker calibration. Classical models, particularly MLP, excel in calibration, producing reliable probability estimates suitable for risk stratification, but often at the cost of lower classification performance due to increased false positives at high sensitivity.

These differences likely arise from architectural and optimization distinctions. QNNs variational quantum circuits may leverage complex feature interactions, potentially via entanglement, to enhance classification, but simulated noise (e.g., depolarizing errors) and stochasticity from multiple runs can impair probability calibration. Conversely, classical models like MLP, optimized for log loss minimization, prioritize well-calibrated probabilities, though lower decision thresholds at 83% sensitivity reduce their specificity and PPV.

Clinically, QNNs are promising for screening anastomotic leak due to robust classification performance, while classical models like MLP are better suited for precise risk assessment. The findings provide valuable insights into the practical implementation of QNNs, guiding the selection of ansatz-optimizer pairs to achieve the desired balance of performance, robustness, and reliability in post-surgical complication detection. Future work could explore post-hoc calibration techniques for QNNs or hybrid quantum-classical approaches to combine the classification strength of quantum approaches with the calibration reliability of classical methods.

### Anastomotic leak risk factors analysis

Although logistic regression provides a well-established and interpretable framework for understanding feature effects through odds ratios, QNN feature importance analyses (permutation-based and gradient-based) are novel and less validated, serving as an exploratory approach to uncover complex patterns in the decision-making process of the quantum model.

The logistic regression model developed to predict the probability of anastomotic leak is expressed as

$$\begin{aligned} \ln \left( \frac{p}{1-p}\right)&= 1.436 - 1.149 \cdot \text {DM} \\&\quad - 1.429 \cdot \text {Smoking} \\&\quad - 0.952 \cdot \text {ACSP} \\&\quad - 1.610 \cdot \text {NoCoil}, \end{aligned}$$

where *p* represents the probability of an anastomotic leak, and the variables DM (Diabetes Mellitus), Smoking, ACSP (use of a specific treatment method), and NoCoil (non-use of a rectal tube) are dichotomous predictors. The point estimates of the odds ratios (OR) with their corresponding 95% confidence intervals (CI) for each variable are given by

$$\begin{aligned} \text {OR(DM)}&= e^{-1.149} = 3.16 \, (95\% \, \text {CI}: 1.17, 8.32), \\ \text {OR(Smoking)}&= e^{-1.429} = 4.18 \, (95\% \, \text {CI}: 1.51, 11.48), \\ \text {OR(ACSP)}&= e^{-0.952} = 2.59 \, (95\% \, \text {CI}: 0.92, 8.75), \\ \text {OR(NoCoil)}&= e^{-1.610} = 5.00 \, (95\% \, \text {CI}: 1.47, 23.82). \end{aligned}$$

For dichotomous variables, the odds ratio compares the odds of the outcome (Anastomotic Leak) in the presence versus the absence of the risk factor. In this model, the riskier category for DM and Smoking is coded as 1, while for ACSP and NoCoil, the riskier category is coded as 0 (indicating the absence of these treatment methods). Thus, patients with diabetes have approximately 3.16 times higher odds of experiencing an anastomotic leak compared to those without diabetes. Similarly, smokers have approximately 4.18 times higher odds. The absence of the ACSP treatment method increases the odds of a leak by approximately 2.59 times. Notably, patients without a rectal tube (NoCoil = 0) have approximately 5.00 times higher odds of an anastomotic leak compared to those with the tube, underscoring the protective effect of this intervention.

The probability of an anastomotic leak can be calculated using the equation

$$\begin{aligned} P(\text {LEAK} = 1)&= \frac{\exp (1.436 - 1.149 \cdot \text {DM} - 1.429 \cdot \text {Smoking} - 0.952 \cdot \text {ACSP} - 1.610 \cdot \text {NoCoil})}{1 + \exp (1.436 - 1.149 \cdot \text {DM} - 1.429 \cdot \text {Smoking} - 0.952 \cdot \text {ACSP} - 1.610 \cdot \text {NoCoil})}. \end{aligned}$$

These findings highlight the significant impact of smoking and the non-use of a rectal tube as key risk factors for anastomotic leak, providing a robust classical baseline for identifying high-risk patients.

**Fig. 11:**
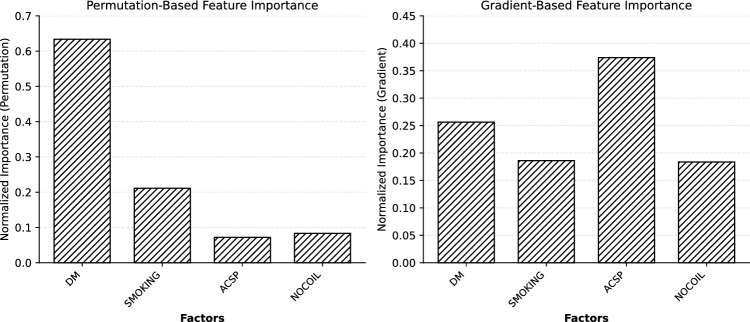
Comparison of normalized feature importance scores for predicting anastomotic leak. The permutation analysis (left) highlights the dominant global influence of DM, while the gradient-based analysis (right) emphasizes the localized sensitivity to ACSP.

In contrast to the additive log-odds approach of classical regression, the QNN feature importance analysis reveals distinctly different patterns (Fig. 11). The permutation-based importance analysis (Fig. 11, left), which assesses the global impact of variables on the model’s AUC, demonstrates that DM is the most influential predictor with a normalized score of 0.6373. While this aligns with the logistic regression results, the QNN heavily amplifies its relative importance over Smoking (0.1890), NoCoil (0.1042), and ACSP (0.0695).

The gradient-based importance analysis (Fig. 11, right), which measures the model’s localized sensitivity to feature perturbations, provides another distinct perspective. Here, ACSP emerges as the most critical feature with an importance score of 0.3739, a dramatic shift from its minimal role in both the permutation analysis and logistic regression. DM maintains substantial influence (0.2564), while Smoking (0.1860) and NoCoil (0.1836) demonstrate nearly equivalent contributions.

Ultimately, the key result of this interpretability analysis is the striking divergence in feature rankings. While classical logistic regression identifies Smoking and NoCoil as the most dominant predictors based on linear odds, the QNN analyses highlight DM and ACSP. This divergence indicates that the QNN does not simply replicate classical linear associations, but instead captures complex, non-linear dependencies and feature interactions through its quantum entangled circuit structure.

### Summary of key findings

Our evaluations of QNNs trained by different optimizers using two different ansatze against hyperparameter-tuned classical models yielded several insights for predicting anastomotic leak.

QNNs demonstrated robust discriminative power in handling the dataset’s inherent class imbalance. When decision thresholds were optimized for a clinically necessary high sensitivity (83%), a distinct performance divergence emerged between the two computational paradigms. Classical models struggled with high false-positive rates at this threshold, achieving a maximum specificity of only 44% (Gaussian Naive Bayes).

In contrast, specific QNN configurations successfully minimized false positives while maintaining the required detection rate, achieving significantly higher specificities, such as 66% (EfficientSU2-BFGS) and 64% (RealAmplitudes-CMA-ES). Because sensitivity was fixed, this robust threshold-dependent classification makes the QNN architectures highly effective at correctly identifying true negatives and minimizing false alarms in clinical screening.

The analysis also revealed a distinct trade-off regarding probability calibration. Classical models, particularly the MLP, exhibited superior calibration metrics, characterized by lower Brier scores (0.103) and Log Loss (0.359). Therefore, while QNNs excelled at the binary separation of risk classes, classical models remain currently more reliable for continuous risk stratification.

Finally, our results underscore the critical role of optimizer selection in navigating the complex, stochastic loss landscapes of variational quantum circuits. We established a direct link between the resilience of an algorithm to noise and the ultimate predictive power of QNNs. The theoretical noise-resilience of evolutionary strategies like CMA-ES^84^ translates directly to our empirical findings, enabling high specificity and stable convergence despite the presence of simulated hardware noise and finite sampling. While gradient-based methods like BFGS achieved highly competitive final errors within this specific simulation, the derivative-free stability of CMA-ES across independent runs confirms its utility as a reliable, robust tool for training quantum classifiers as models scale toward realistic clinical environments.

## Discussion

The application of Quantum Neural Networks to clinical risk prediction represents an alternative computational approach, but it also necessitates a careful evaluation of practical trade-offs. In this section, we contextualize the experimental results from a strictly medical perspective, weighing the distinct advantages of quantum screening against the reliable probability calibration of classical models. We also critically assess the limitations of the current study, defining the necessary steps for future validation and responsible clinical deployment.

### Clinical implications

The findings of this comparative analysis bear significant implications for the application of quantum machine learning in biomedical contexts, particularly for critical tasks like the early detection of rare post-surgical complications. The QNNs demonstrated strength in threshold-based classification, particularly its superior performance in metrics pertinent to high sensitivity, suggests its potential as a tool for screening and identifying patients at risk of anastomotic leak.

In settings where minimizing missed cases (false negatives) is paramount, a defining characteristic of rare and severe complication detection, the QNNs classification profile offers a measurable advantage. However, the generalizability of these results is constrained by the study’s limited sample size (N=200, with 28 anastomotic leak events), which inherently reduces statistical power and increases overfitting risk. While rigorous feature selection and cross-validation mitigate this, external validation in larger, multi-center cohorts is essential before clinical deployment.

In contrast, the MLP proficiency in probability calibration positions it as a valuable asset in scenarios where accurate risk stratification and reliable probability estimates are central. For example, in clinical decision-making processes that rely on nuanced risk assessments to guide personalized interventions, the well-calibrated probabilities provided by MLP could be highly beneficial.

The trade-off we identified has direct clinical relevance. A high QNN score, indicating a high risk of anastomotic leak, could serve as an effective clinical decision support alert. For example, it could automatically trigger a recommendation for closer post-operative monitoring, a delayed oral diet, or a follow-up CT scan for at-risk patients. The high NPV of the top-performing QNNs (up to 96%) is particularly valuable, providing strong confidence in identifying patients who are unlikely to develop a leak. Conversely, the well-calibrated probability from an MLP could be integrated into a broader patient risk score, helping clinicians balance the risk of anastomotic leak against other surgical risks when planning the intervention itself. Even a modest improvement in NPV from 93% (MLP) to 96% (QNN) means that for every 100 patients predicted to be low-risk, three fewer will have been incorrectly classified, representing a meaningful gain in patient safety.

### Discussion on sample size and generalizability

It is critical, however, to contextualize these promising findings within the primary limitation of this study: the small sample size (N=200, with 28 anastomotic leak events). While we employed rigorous feature selection from the initial variable pool and extensive cross-validation to mitigate overfitting, the statistical power remains limited. This inherently constrains the generalizability of our findings and demands caution in their interpretation. The impressive performance metrics, particularly the high NPV and accuracy of the QNNs, must be considered preliminary. The small number of positive events (anastomotic leak occurrences) presents a significant challenge for any machine learning model, especially for a QNN intended to learn complex, non-linear relationships and quantum correlations. Future work must prioritize the validation of these models on larger, independent, and ideally multi-center datasets. Only through such large-scale validation can the true clinical utility and potential advantages of QNNs shown here be definitively established and considered for any clinical application.

## Conclusion

Our central finding is a distinct trade-off between classification power and probability calibration when evaluating QNNs against hyperparameter optimized classical models. At a fixed, clinically crucial sensitivity of 83%, QNNs simulated with realistic hardware noise demonstrated robust classification capabilities. Specific QNN configurations achieved significantly higher specificity (up to 66%) and Negative Predictive Value (up to 96%) compared to classical models (maximum 44% and 94%, respectively) at this high sensitivity threshold, effectively minimizing false positives. Conversely, classical models, particularly the Multi-Layer Perceptron, excelled at probability calibration, making them better suited for continuous risk stratification.

A significant contribution of this work is demonstrating the direct link between the efficacy of the chosen optimization algorithm and the final predictive power of the QNN. Evolutionary approaches like CMA-ES proved particularly robust in the optimization of variational parameters against simulated shot noise and gate errors, offering stable performance across independent runs.

While these findings must be contextualized within the primary limitation of the study, a small clinical cohort ($N=200$, with 28 anastomotic leak events), our feature inclusion was strictly bounded a priori by surgical realities and the availability of temporal data to aggressively minimize overfitting alongside extensive cross-validation. Although the high expressive capacity of parameterized quantum circuits necessitates that these potential advantages be validated on larger, independent datasets, this study establishes the tangible utility of QNNs for clinical screening. Ultimately, our work underscores the necessity of evaluating both classification threshold metrics and probability calibration to select the most appropriate computational tool for specific medical risk assessment tasks.

## Figures and Tables

**Fig. 12:**
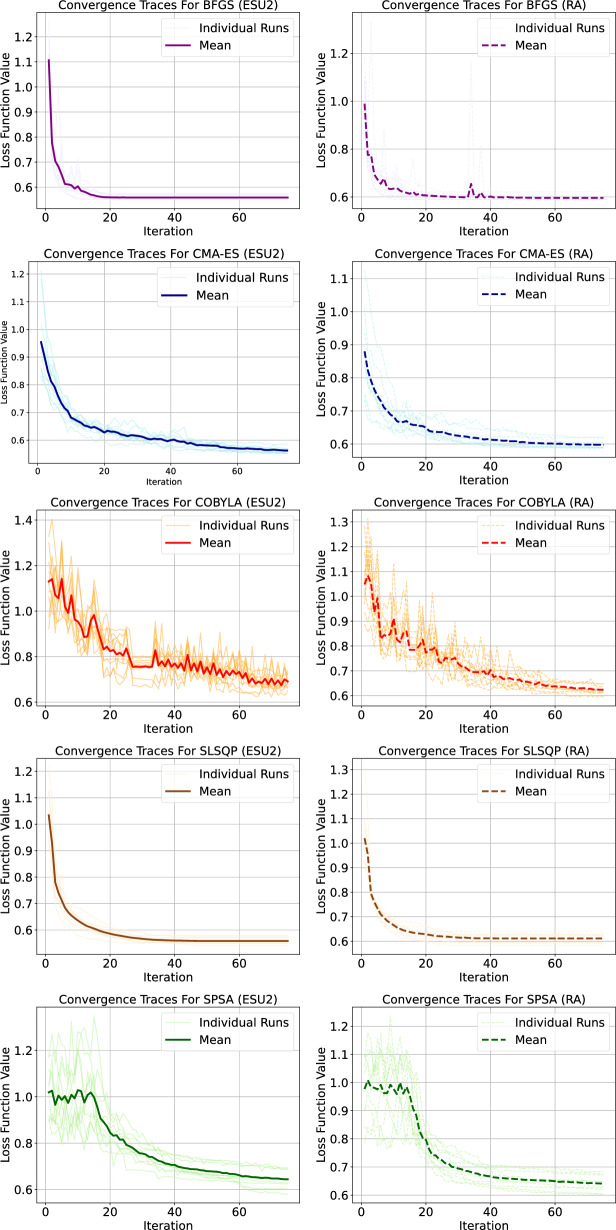
Individual convergence traces over 10 independent runs for the evaluated optimizers. The left column displays optimization for the EfficientSU2 (ESU2) ansatz, while the right column displays the RealAmplitudes (RA) ansatz.

**Fig. 13:**
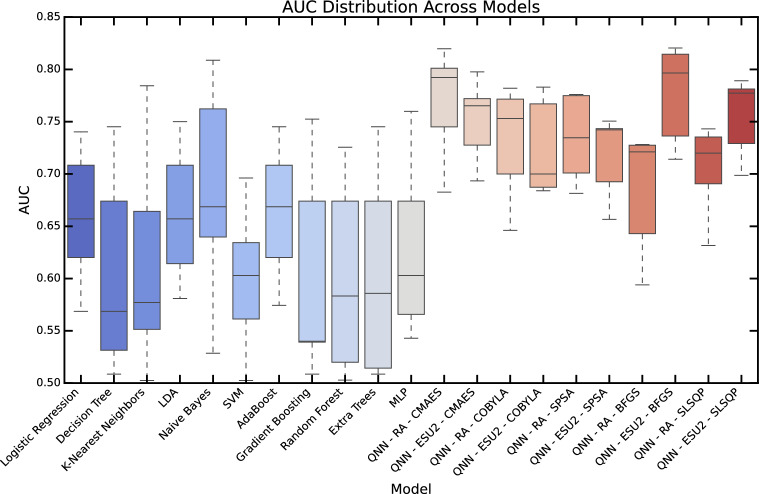
Boxplot of classification model performance across cross-validation folds. The distribution of AUC is shown for each model, illustrating variability and robustness to different training data subsets.

**Fig. 14:**
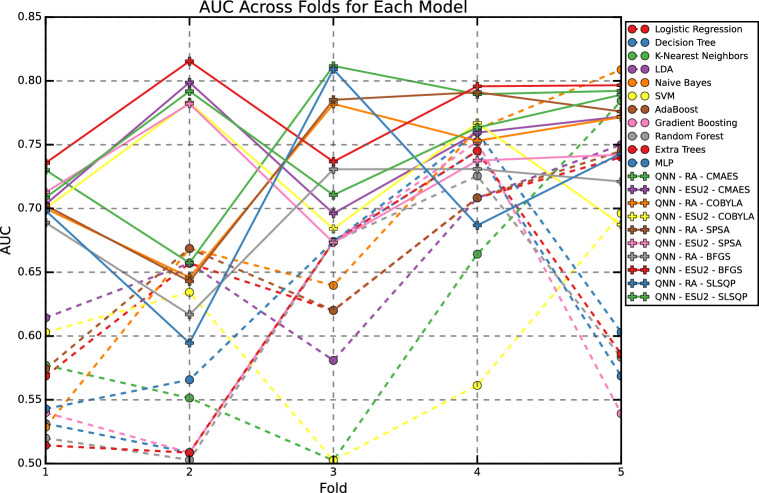
Line plot showing classification model performance across cross-validation folds. Each point represents a fold, and the trend lines indicate how models perform on different training subsets.

**Fig. 15:**
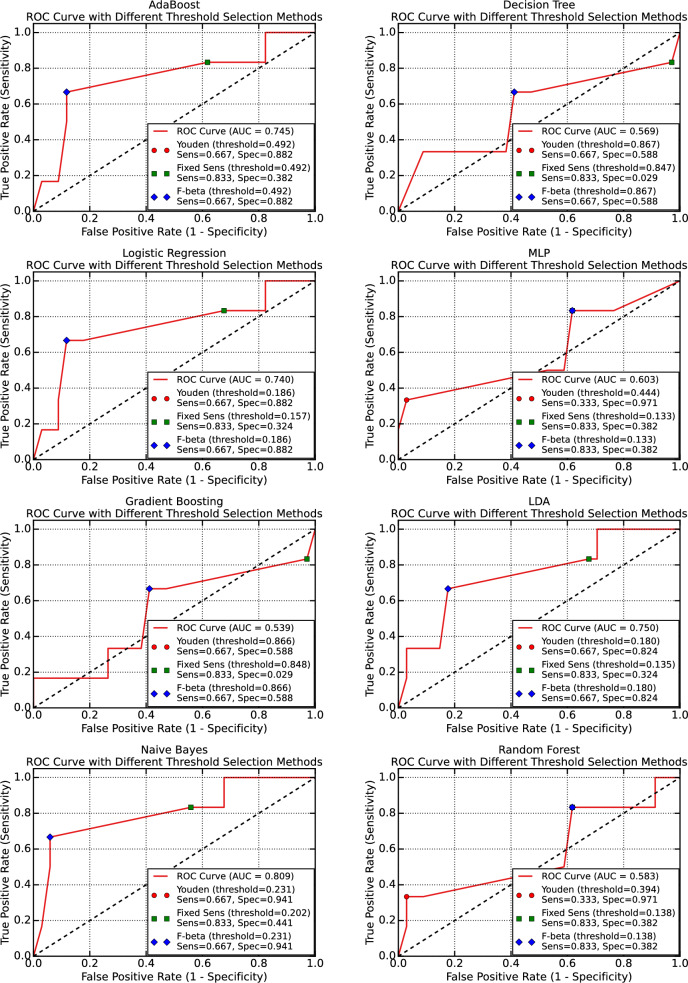
ROC curves for the 11 classical machine learning models evaluated on the fifth cross-validation fold.

**Fig. 16:**
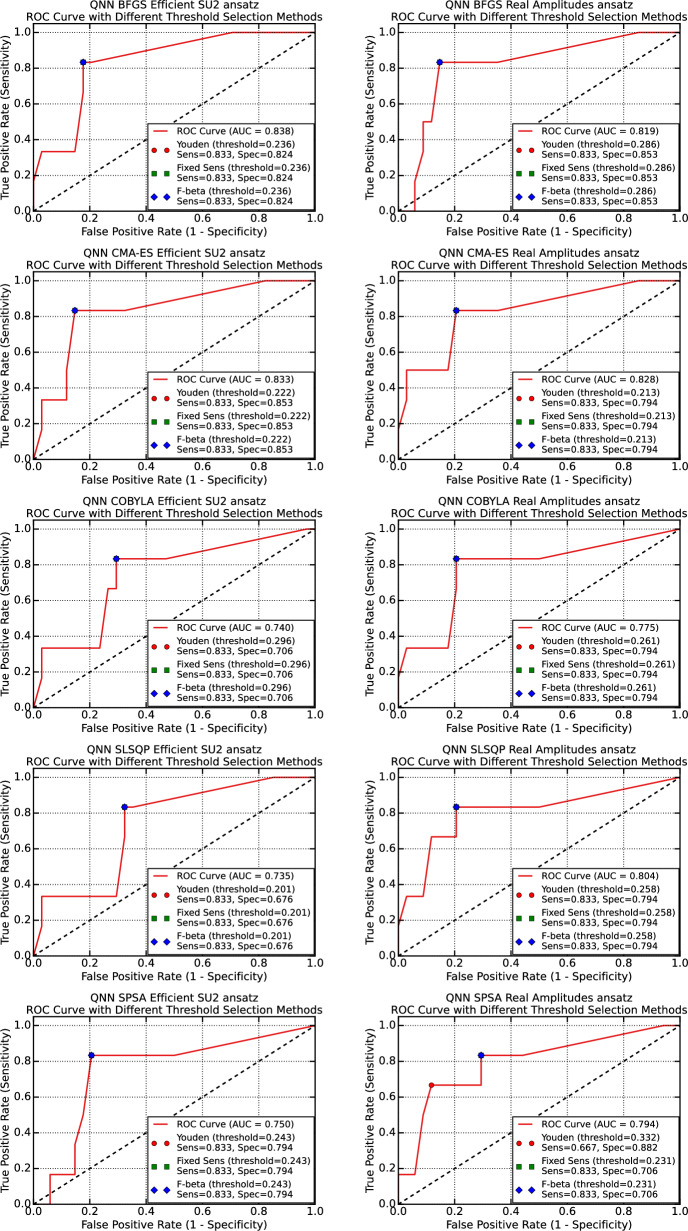
ROC curves for the 10 Quantum Neural Network (QNN) models evaluated on the fifth cross-validation fold.

## Data Availability

The datasets generated and analyzed during the current study, after appropriate anonymization to protect patient privacy, are available from the corresponding author upon reasonable request. The source code for simulating noise within the Variational Quantum Classifier (VQC) framework is publicly available at https://github.com/VojtechNovak/qnn_leak.

## Appendix A Individual runs of optimizers

This appendix section provides the individual convergence curves for ten distinct optimization runs for each optimizer-ansatz combination. These plots visually demonstrate the stability and run-to-run variability of various optimizers when applied to the different quantum circuit ansatzes. For each set of runs, a mean convergence line is also included to facilitate comparison with the aggregated plots presented in Fig. 12.

## Appendix B Cross validation

Our cross-validation approach involved partitioning the dataset into multiple folds. In each iteration, a different fold was held out as the test set, while the remaining folds were used for training. This process was repeated until each fold had served as the test set exactly once. We quantified model performance in each fold using the Area Under the ROC Curve, a metric that reflects the overall discriminatory power of the model across various thresholds.

To visualize the cross-validation results, we present both a boxplot (Fig. 13) and a line plot (Fig. 14). The boxplot (Fig. 13) summarizes the distribution of AUC scores across all folds for each model. This visualization effectively illustrates the central tendency and variability of each model’s performance across different training subsets. A tighter box in the boxplot indicates more consistent performance and greater robustness.

The line plot (Fig. 14) provides a complementary perspective by displaying the AUC score for each fold individually. This allows for the visualization of performance fluctuations across different training datasets and helps in identifying any potential instability or high variance in model performance.

The cross-validation results, as depicted in Figs. 13 and 14, demonstrate the consistent performance of the Quantum Neural Network (QNN) with the RealAmplitudes ansatz. The boxplot (Fig. 13) clearly shows that the QNN consistently achieves higher median AUC scores compared to classical models such as Logistic Regression and Gradient Boosting, suggesting a superior average performance across different data partitions. Furthermore, the line plot (Fig. 14) indicates that while some performance variation exists across folds for all models (attributable to the inherent variability in training data subsets), the QNN maintains a relatively stable and high-performing trajectory across these variations. This robustness across different cross-validation folds reinforces the generalizability of the QNN and its potential for reliable predictive performance on unseen patient data, setting it apart from classical models that exhibit either lower average performance or greater performance variability, as observed in the box and line plots.

## Appendix C Future work

This study provides a foundational step in exploring the application of QNNs for post-operative complication prediction using classical simulations. To rigorously validate the potential clinical utility of QNNs and to fully leverage the capabilities of quantum computation, future research will focus on several key directions:

- *Quantum hardware implementation:* The immediate next step is to transition from classical simulations to experiments on actual quantum computing hardware. This is crucial to assess the real-world performance of QNNs and to explore whether they can maintain or enhance their predictive power when executed on quantum devices. These experiments will involve deploying the developed QNN models on available quantum platforms and systematically evaluating their performance in practical quantum computing environments.
- *Impact of quantum noise and hardware constraints:* While this study incorporated simulated noise models, including depolarizing errors and sampling noise, future work will extend these investigations to real quantum hardware. Experiments on quantum devices will inherently introduce additional noise sources, fidelity errors, limited coherence times, and connectivity constraints. Future studies will explicitly characterize how these hardware limitations affect QNN performance, with a focus on developing noise-resilient quantum circuits and error mitigation techniques tailored to biomedical prediction tasks.
- *Exploration of quantum advantage:* As quantum hardware matures, future studies will aim to identify specific scenarios or model configurations where QNNs can demonstrably outperform state-of-the-art classical methods, establishing a tangible quantum advantage in biomedical classification. This will require careful benchmarking against optimized classical algorithms and architectures, alongside rigorous statistical validation to confirm any observed quantum speedup or accuracy improvements.
- *Advanced QNN architectures and training techniques:* Future research will explore more advanced QNN architectures, including alternative ansatzes (e.g., hardware-efficient or problem-inspired ansatzes) and feature maps (e.g., higher-order or data-adaptive feature maps), to further optimize performance for clinical prediction tasks. Additionally, hybrid quantum-classical training methodologies and techniques to improve the calibration of QNN probability estimates will be investigated, potentially bridging the gap between QNN classification strength and MLP calibration reliability.

These future directions are essential to move beyond classical simulations and towards a realistic assessment of QNNs potential for transforming clinical diagnostics and predictive medicine, ultimately paving the way for practical quantum-enhanced tools in healthcare.

## Appendix D Model descriptions and hyperparameter optimization

To evaluate a range of classification algorithms, ten machine learning models were employed: Logistic Regression, Decision Tree, KNN, Linear LDA, Naive Bayes, SVM with a radial basis function kernel, AdaBoost, Gradient Boosting, Random Forest, and Extra Trees.

The dataset was split into five folds for outer cross-validation to ensure robust evaluation. For each fold, the training data was used to perform a 3-fold inner cross-validation grid search to identify the optimal hyperparameters for each classifier, maximizing the AUC score. The target variable, originally encoded as $\{-1, 1\}$, was transformed to $\{0, 1\}$ using a label encoder, and features were standardized using a standard scaler to have zero mean and unit variance. After optimization, the best model for each classifier was evaluated on the test set of each fold. If the AUC score was below 0.5, the predicted probabilities were flipped (i.e., $p \rightarrow 1-p$) to correct for potential label misalignment. The optimal classification threshold was determined by maximizing the F1-score on the test set.

Table 10 summarizes the models, their hyperparameter search spaces, and the optimal configurations found across the five folds.

**Table 10:** Model descriptions, grid search spaces, and optimal hyperparameters.

Classifier	Description & search space	Optimal hyperparameters (Folds 1–5)
Logistic regression	Linear model using a logistic function. Regularization is applied to prevent overfitting. *Space:* $C \in \{0.01, 0.1, 1, 10\}$, penalty $\in \{\text {L1}, \text {L2}\}$	**Folds 1–4:** $C=1$, penalty=l1 **Fold 5:** $C=1$, penalty=l2
K-nearest neighbors	Non-parametric model based on majority class of *k* neighbors. *Space:* $k \in \{3, 5, 7, 9\}$, weights $\in \{\text {uniform}, \text {distance}\}$	**Fold 1:** $k=3$, weights=distance **Folds 2, 4, 5:** $k=5$, weights=distance **Fold 3:** $k=9$, weights=uniform
LDA	Linear model finding a combination of features to separate classes. *Space:* solver $\in \{\text {svd}, \text {lsqr}\}$	**Folds 1–5:** solver=svd
Naive Bayes	Probabilistic model based on Bayes’ theorem, assuming feature independence. *Space:* Default parameters (Gaussian)	**Folds 1–5:** Default parameters
SVM (RBF Kernel)	Optimal hyperplane separation using an RBF kernel for non-linear boundaries. *Space:* $C \in \{0.1, 1, 10\}$, $\gamma \in \{0.01, 0.1, 1\}$	**Fold 1:** $C=10, \gamma =0.1$ **Fold 2:** $C=0.1, \gamma =0.01$ **Fold 3:** $C=0.1, \gamma =0.1$ **Fold 4:** $C=1, \gamma =1$ **Fold 5:** $C=10, \gamma =1$
AdaBoost	Ensemble combining weak learners (SAMME^85^ algorithm used). *Space:* estimators $\in \{50, 100, 200\}$, lr $\in \{0.01, 0.1, 1\}$	**Fold 1:** lr=1, $n=100$ **Fold 2:** lr=0.1, $n=50$ **Fold 3:** lr=1, $n=50$ **Fold 4:** lr=0.01, $n=100$ **Fold 5:** lr=0.1, $n=200$
Gradient boosting	Sequential tree building minimizing a loss function. *Space:* estimators $\in \{100, 200\}$, lr $\in \{0.01, 0.1\}$, depth $\in \{3, 5\}$	**Fold 1:** lr=0.1, max_depth=3, $n=100$ **Folds 2, 5:** lr=0.01, max_depth=5, $n=100$ **Folds 3, 4:** lr=0.01, max_depth=3, $n=100$

## Appendix E Quantum measurement operators

The quantum neural network classification is performed through projective measurements on the quantum circuit output. This section describes the measurement operators employed in our implementation using the Qiskit Sampler primitive.

Our quantum classifier operates on a 4-qubit system, where measurements are performed in the computational basis $\{|0\rangle , |1\rangle \}^{\otimes 4}$. The measurement operators are defined as the set of projection operators $\hat{M}_s = |s\rangle \langle s|$, where $s \in \{0, 1\}^4$ represents all possible 4-bit computational basis states, and $|s\rangle = |s_0 s_1 s_2 s_3\rangle$ with $s_i \in \{0, 1\}$. The complete set of 16 measurement operators for our 4-qubit system is $\hat{M}_{0000} = |0000\rangle \langle 0000|, \hat{M}_{0001} = |0001\rangle \langle 0001|, \dots , \hat{M}_{1111} = |1111\rangle \langle 1111|$. These operators satisfy the completeness relation $\sum _{s=0}^{15} \hat{M}_s = \hat{I}^{\otimes 4}$, where $\hat{I}$ is the single-qubit identity operator.

For a quantum state $|\psi (\boldsymbol{\theta }, \textbf{x})\rangle$ prepared by the parameterized quantum circuit with parameters $\boldsymbol{\theta }$ and input features $\textbf{x}$, the measurement probabilities are

$$\begin{aligned} p_s = \langle \psi (\boldsymbol{\theta }, \textbf{x})|\hat{M}_s|\psi (\boldsymbol{\theta }, \textbf{x})\rangle = |\langle s|\psi (\boldsymbol{\theta }, \textbf{x})\rangle |^2. \end{aligned}$$

The Qiskit Sampler primitive performs shot-based sampling to estimate these probabilities through repeated measurements. The classification probabilities are computed as $P(\text {class 0}) = \sum _{s: \text {parity}(s) = 0} p_s$ and $P(\text {class 1}) = \sum _{s: \text {parity}(s) = 1} p_s$, where $\text {parity}(s) = s_0 \oplus s_1 \oplus s_2 \oplus s_3$ is the parity of the bit string *s*.

In our implementation using the Variational Quantum Classifier (VQC) from Qiskit Machine Learning, the Sampler primitive handles the measurement process automatically. A default shot count is used for probability estimation (typically 1024 shots), and the predict_proba method returns the two-class probabilities $[P(\text {class 0}), P(\text {class 1})]$. No explicit measurement operators need to be defined in the code as they are handled internally by the VQC framework.

The shot-based sampling introduces statistical uncertainty in probability estimates. For a probability *p* estimated with *N* shots, the standard error is approximately $\sigma _p$ $\approx$ $\sqrt{p(1-p)/N}$. This measurement uncertainty contributes to the overall variance in the quantum neural network performance and is accounted for in our cross-validation methodology.

## Appendix F ROC curves

Model performance was evaluated using Receiver Operating Characteristic curves derived from the fifth cross-validation fold to ensure consistency across benchmarks (Figs. 15 and 16). To assess the trade-offs between sensitivity and specificity, we identified three distinct operating points:

1. *Youden index (**J**):* The threshold that maximizes the sum of sensitivity and specificity, defined as $J = \text {Sensitivity} + \text {Specificity} - 1$. This identifies the point of maximum global diagnostic accuracy on the ROC curve.
2. *Fixed sensitivity (83%):* A constrained threshold set to 83% sensitivity. This provides a standardized comparison of specificity and other metrics at a clinically relevant level for detecting rare post-surgical complications.
3. $F_{\beta }$* Score optimization:* The weighted harmonic mean of precision and recall, allowing for prioritized sensitivity over precision to align with clinical risk-aversion strategies.
